# Fast Fourier Transform Enables Automated Parametrization of Complex Dihedral Potentials in All-Atom and Coarse-Grained Force Fields

**DOI:** 10.1021/acs.jcim.6c00123

**Published:** 2026-05-19

**Authors:** Humberto T. Flores-Trujillo, Guillermo L. Rodríguez-Segura, Carlos Amador-Bedolla, Laura Dominguez

**Affiliations:** † Departamento de Fisicoquímica, Facultad de Química, 164178 Universidad Nacional Autónoma de México, Ciudad de México 04510, México; ‡ Departamento de Física y Química Teórica, Facultad de Química, 7180 Universidad Nacional Autónoma de México, Ciudad de México 04510, México

## Abstract

Torsional parametrization remains one of the most persistent challenges and weaknesses of modern force fields, particularly for dihedrals whose asymmetry and multimodality evade traditional Fourier or Ryckaert–Bellemans treatments [ ShirtsM. R., ; MobleyD. L., In Biomolecular Simulations, Springer, 2013; Vol. 9, pp 71–120 and BowmanJ. M.,  Chem. Rev. 2017, 117, 10034–10072]. Here, we introduce a general and fully automated methodology for dihedral parametrization in both all-atom (AA) and coarse-grained (CG) models based on the Fast Fourier Transform (FFT). This Fourier-analysis framework provides a systematic and unbiased route to reconstruct torsional energy profiles of arbitrary complexity, including nonsymmetric and multimodal shapes that have remained inaccessible to existing parametrization tools. When combined with iterative refinement via QM–MM energy matching in AA models and Iterative Boltzmann Inversion in CG models, our FFT approach yields torsional potentials that quantitatively reproduce reference energy landscapes across a wide variety of chemical environments. We apply this methodology to different molecules within an AA framework, obtaining consistently improved agreement with QM reference profiles. In the CG regime, our method is demonstrated on two systems, the MS-Z molecular switch and the Aβ42 peptide, yielding transferable torsional potentials that enable accurate modeling of their conformational behavior. Overall, this work demonstrates that a FFT-based torsional parametrization is a robust and general strategy for developing next generation force fields.

## Graphical abstract

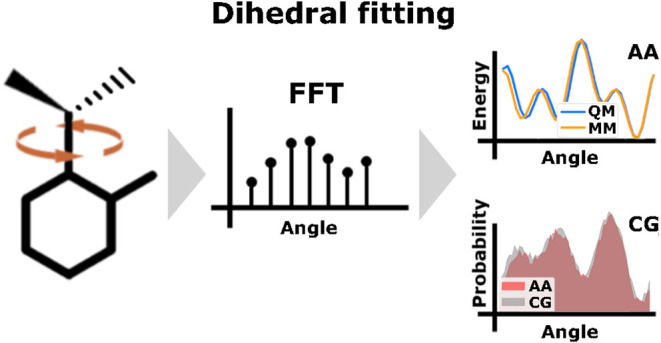

## Introduction

Molecular dynamics (MD) simulations rely on force fields whose accuracy depends critically on the quality of their underlying parameters.^1^ Consequently, parameter selection and refinement are essential, as they directly govern conformational sampling and, ultimately, the fidelity of a simulation. Among these parameters, torsional terms play a central role because they describe rotations around covalent bonds. These rotations define the accessible conformational landscape and therefore determine molecular flexibility and dynamics. In proteins, torsions are a key component of their secondary structure, flexibility, and function.^3^ Similarly, in small molecules such as biphenyl, torsional angles around the inter-ring bond govern atropisomerism and rotational barriers that profoundly influence molecular properties and interactions^4^ and in Feringa’s molecular motors, torsional angles control unidirectional rotary motion through precisely tuned energy barriers, enabling light-driven mechanical work.^5^ Despite significant advances in automated parametrization tools for both all-atom (AA) and coarse-grained (CG) models,^6–10^ a robust and universally applicable methodology for dihedral parametrization is still lacking.^11,12^ The challenges stem from the complexity of torsional energy profiles, which often feature multiple minima and pronounced asymmetry arising from rotational barriers, as well as from the four-atom definition of dihedral angles that engenders coupled interactions, structural variability, and heightened susceptibility to steric clashes.^13,14^ These factors reduce parameter transferability and make torsional terms considerably more difficult to parametrize than bonds, angles, or nonbonded interactions, particularly within automated workflows that rely on simplified fitting strategies.

Current parametrization tools either neglect torsional fitting altogether or restrict their treatment to symmetric torsions, leaving asymmetric cases that arise from chiral centers, heteroatomic substitutions, or sterically crowded environments insufficiently described.^15^ As a result, the absence of a general and efficient approach to torsional parametrization remains one of the principal limitations in automated force-field development.^16,17^ Here we demonstrate that the Discrete Fourier Transform (DiFT), a robust and computationally efficient Fourier-based decomposition, provides a general and automatic solution to this problem.

Most traditional force fields, including the General AMBER Force Field (GAFF2),^18,19^ GROMOS,^20^ OPLS-AA,^21^ and CHARMM,^22^ employ a truncated Fourier series to represent the torsional potential associated with a given dihedral angle.^2,23^ This functional form affords considerable flexibility, enabling the reconstruction of energy landscapes that contain multiple minima and maxima. The torsional potential is typically expressed as

1 $$V_{\mathrm{ijkl}}^{F}\left(n,\phi \right)=\sum_{n}^{N}A_{n}\left[1+\cos\left(n\phi -\omega_{n}\right)\right]$$

where *n* denotes the multiplicity (or frequency), *A*_*n*_ is the amplitude (or force constant), and ω_*n*_ represents the phase shift of the corresponding term. An alternative, and widely adopted, formulation is the Ryckaert–Bellemans (RB) potential,^24^ a fifth-order polynomial in cos ϕ, defined as

2 $$V_{\mathrm{ijkl}}^{\mathrm{RB}}\left(n,\phi \right)=\sum_{n=0}^{5}R_{n}\cos^{n}\left(\phi \right)$$

Both the Fourier and RB functional forms allow for the description of torsional energy surfaces with multiple maxima and minima. Despite the diversity of available torsional potentials, the key difficulty remains in the selection of an appropriate model and the determination of its parameters. To achieve this, the standard procedure involves performing potential energy scans along the dihedral of interest using both quantum-mechanical (QM) and molecular-mechanical (MM) calculations.^6,14^ These scans yield an energy profile that enables refinement of the MM torsional term so that it reproduces the QM reference within the chosen force-field framework:

3 $$V_{\mathrm{QM}}=V_{\mathrm{MM}}+V_{\mathrm{tors}}$$

The form of the torsional potential *V*_tors_ is dictated by the structural characteristics of the dihedral angle under consideration. For example, dihedrals connecting two aromatic rings typically yield a symmetric, multimodal profile. A similar behavior is often observed for dihedrals between adjacent carbons in an aliphatic chain. To reproduce such profiles using one of the functional forms described above, several parametrization strategies exist, with Linear Least-Squares (LLS) regression being the most widely employed.^14,25^ This procedure provides the required coefficients according to eq 2.

Important complications arise, however, when the torsional energy profile is asymmetric, such as when the dihedral lies in the vicinity of a chiral carbon. In these cases, the lack of spatial symmetry leads to intrinsically asymmetric energy profiles, as rotations in opposite directions are not energetically equivalent. This asymmetrical behavior reflects the underlying multidimensional potential energy surface rather than a limitation of the torsional representation. In these cases, fitting standard torsional potential forms becomes considerably more challenging.^26,27^ Several methodologies have been proposed to address this issue, most of which focus on determining the appropriate phase values ω_*n*_ in eq 1. These approaches typically rely on optimization schemes, heuristics, or minimization routines, often at substantial computational cost.^27–30^ Monte Carlo–based strategies incorporating simulated annealing have also been explored.^31^ Hopkins further demonstrated that an LLS approach can successfully reproduce asymmetric torsional profiles, as shown for the dihedral in amoxicillin using a four-term Fourier series.^14^

Although several methodologies have been proposed to reproduce asymmetric torsional profiles, they are often computationally expensive, difficult to reproduce, or restricted to functional forms with a limited number of terms. For this reason, we introduce a new Fourier-based technique that enables the automatic determination of the coefficients in eq 1 for any torsional energy profile, whether symmetric or asymmetric, or even symmetric profiles that cannot be represented by RB-type potentials. This approach relies on the DiFT,^32^ which takes an evenly spaced set of discrete data and generates a frequency-domain representation whose real part corresponds to a sum of cosine terms suitable for reconstructing torsional potentials. The central equation of the DiFT formalism is

4 $$X_{n}=\sum_{k=0}^{M-1}x_{k}\left[\cos\left(-\frac{2\pi \mathrm{nk}}{M}\right)+i\sin\left(-\frac{2\pi \mathrm{nk}}{M}\right)\right]$$

where *x*_*k*_ denotes the set of *M* equally spaced samples of a function *f* over the interval [0,2π). The amplitude *A*_*n*_ and phase ω_*n*_ associated with the *n*-th frequency component *X*_*n*_ can be obtained from its real and imaginary parts with

5 $$A_{n}=\frac{2}{M}\sqrt{R{\left(X_{n}\right)}^{2}+I{\left(X_{n}\right)}^{2}}$$

6 $$\omega_{n}=\arctan\left[\frac{I\left(X_{n}\right)}{R\left(X_{n}\right)}\right]$$

eqs 4–6 can be computed for *n* = 0, 1, ..., Nyquist, where the Nyquist limit allows us to avoid redundant frequencies. This limit should be determined based on the size of the input data set *x*_*k*_ as follows

$$\mathrm{Nyquist}=\frac{M}{2},\left(\mathrm{when}\;M\;\mathrm{is}\;\mathrm{even}\right)$$

$$\mathrm{Nyquist}=\frac{M-1}{2},\left(\mathrm{when}\;M\;\mathrm{is}\;\mathrm{odd}\right)$$

In principle, the DiFT method allows the generation of as many Fourier components {*X*_*n*_} as there are data points {*x*_*k*_}. However, when the Nyquist limit is considered, only components up to the Nyquist frequency are physically independent. It is therefore important to consider the number of data points required as a function of the maximum frequency *N* for which the amplitudes *A*_*n*_ and phases ω_*n*_ are to be determined.

Additionally, when calculating the phase ω_*n*_ using the arctan function, special care must be taken to correctly account for quadrant information, which depends on the working interval. The appropriate conversion between angular units (radians or degrees) must also be performed.

This expression yields the set of Fourier components (*k*) with frequencies *n*_*k*_ where the cosine terms in eq 7 originate from the real part of the DiFT coefficients, according to

$$f\left(\phi \right)=\overset{N}{\underset{n=0}{\sum }}A_{n}\cos\left(n\phi -\omega_{n}\right)$$ 7

Although eqs 1 and 7 are not formally identical, the objective of this work is to assess whether the DiFT-derived coefficients can be used to parametrize the functional form in eq 1.

Fourier-based methods have been employed for decades across a broad range of scientific and engineering disciplines. The DiFT, in particular, was originally developed for the analysis of radio signals but has since found applications in diverse contexts.^2,23,33^ It is a computationally efficient technique that can be readily extended to the analysis and parametrization of torsional potential functions.

To compute eq 4, the Fast Fourier Transform (FFT) can be employed. This approach evaluates the *X*_*n*_ coefficients of the DiFT with a computational complexity of O(M⁡log⁡M) , instead of the $O\left(M^{2}\right)$ scaling required when explicitly evaluating eq 4.^34^ Numerous algorithms exist for implementing FFT, but their diversity and complexity are beyond the scope of this work, so we focus on calculating *X*_*n*_ using an existing dedicated FFT function. Several libraries in different programming languages provide dedicated functions for computing FFTs. Regardless of the method used to obtain the *X*_*n*_ coefficients, the calculation of the amplitudes *A*_*n*_ and phases ω_*n*_ remains the same, following eqs 5 and 6, respectively.

To date, the methodologies used for molecular parametrization, whether for all-atom models (ATB,^35^ LigParGen,^36^ QFORCE^25^) or coarse-grained models (PyCGTool,^37^ Swarm-CG,^38^ Bartender^15^), either do not include a procedure for fitting torsional potentials or restrict it to symmetric dihedrals.

Here, we introduce a general and fully automatic torsional parametrization method based on a Fourier-series decomposition implemented via the Fast Fourier Transform. In contrast to existing approaches, it accurately treats both symmetric and asymmetric torsions, requires no dedicated optimization schemes, and integrates naturally into automated parametrization workflows for atomistic force fields, with the added advantage of being readily transferable to coarse-grained models.

The present work focuses on the parametrization of effective one-dimensional torsional potentials, a practical and widely employed approximation, particularly in coarse-grained models and in cases where the torsional degree of freedom is only weakly coupled to other internal coordinates. However, we must point out that many molecular systems exhibit strong coupling and multiple competing conformations. In such cases a one-dimensional representation may not fully capture the underlying multidimensional energy landscape. The framework introduced here establishes a general route to extend torsional parametrization beyond one dimension, opening the way toward more complete and scalable descriptions of complex conformational behavior.

## Methods

In this work, the methodology was divided into two complementary parts. (1) The first part evaluated the FFT as a general procedure for generating torsional potentials in AA models, using quantum-mechanical and molecular-mechanical potential energy surface scans (PES) as reference data. (2) In the second part, we extended the FFT workflow to the parametrization of torsional angles in CG models, where torsional parametrization is substantially more challenging because a well-defined PES scan cannot be obtained for the well-known atomistic rotatable bonds.^39,40^ In CG models, PES scans are not straightforwardly defined because the reduced representation integrates out atomistic degrees of freedom, resulting in effective interactions that depend implicitly on multiple underlying configurations rather than a single well-defined set of coordinates. To address this limitation, we introduced a novel refinement strategy in which the FFT-derived torsional potentials in the coarse-grained model are optimized using Iterative Boltzmann Inversion (IBI),^41,42^ allowing torsional terms to be refined directly from CG structural distributions while targeting the corresponding atomistic profiles. To our knowledge, this is the first implementation of a combined FFT and IBI framework capable of parametrizing asymmetric torsional potentials.

In both cases, whether from PES scans in atomistic models or from IBI in coarse-grained representations, a target torsional potential *V*_tors_(ϕ_*k*_) is obtained for the dihedral angles under study. The goal of our approach is to construct an analytical representation of this target potential. We determine the Fourier coefficients (*A*_*n*_) and (ω_*n*_) using the FFT. The resulting potential *V*_tors_^FFT^(n,ϕ) provides a continuous description over the full angular domain, while retaining only a reduced set of relevant frequencies.

8 $$V_{\mathrm{tors}}\left(\phi_{k}\right)≅V_{\mathrm{tors}}^{\mathrm{FFT}}\left(n,\phi \right)$$

Given that this work aims to probe the limits of the FFT method and to compare it with existing approaches for describing torsional potentials, the selection of molecules—as well as the parametrization settings—was guided primarily by the shape and complexity of the resulting torsional energy profiles. For this reason, different QM levels of theory and basis sets were used, along with multiple force fields for the MM calculations and MD simulations. Our methodology remains fully independent of these choices and can be seamlessly integrated into diverse parametrization workflows.^43,44^

To select the molecules used in this study, we required that each system contain rotatable dihedral angles whose torsional profiles could be classified into three categories: symmetric, asymmetric, and complex symmetric. To cover a broad range of chemical environments, we examined molecules from different classes, including pharmaceuticals, biomolecules, and organic compounds relevant to solar cell materials.

All torsional angles were modeled using the target functional form defined in eq 1. In addition, an iterative refinement procedure was implemented, analogous to those used in existing parametrization tools, as described below.^25^ All QM calculations were performed with Gaussian 16,^45^ and all MM calculations and molecular dynamics simulations were conducted using GROMACS 2021.^46^ In GROMACS, torsional potentials were implemented using function type 9.

To perform the required Fast Fourier Transform calculations, the FFT module from the NumPy library was used. The all-atom dihedral fitting procedure was implemented with the program dihefit_fft.py, for which detailed usage instructions are provided in the Supporting Information.

### All-Atom Torsional Parametrization Using FFT

First, we assessed the performance of the FFT for reproducing torsional energy profiles of varying symmetry and complexity in AA models. The objective was to determine whether FFT yields accurate coefficients for the torsional potential in eq 1, enabling its direct incorporation into classical force fields. For this, we used the set of molecular fragments described below, which collectively span symmetric, asymmetric, and complex symmetric torsional profiles. The FFT-derived parametrizations were benchmarked against two established approaches commonly employed according to torsional symmetry: (i) the Ryckaert–Bellemans polynomial form for symmetric multimodal profiles, and (ii) Linear Least Squares fitting for asymmetric torsions.

It is important to note that, although an adjustment to an RB-type potential can be performed using LLS, this approach is restricted to symmetric torsional profiles, for which the phase angles ω_*n*_ in eq 1 are limited to 0° or 180°. In contrast, asymmetric torsional profiles require a more general fitting framework that explicitly accounts for nonzero phase shifts. Accordingly, in this work, we use the term LLS exclusively to refer to the general least-squares fitting procedure, while emphasizing that its application to RB-type potentials is inherently limited to symmetric cases.

#### Fragmentation Scheme for QM and MM Scans

To obtain accurate torsional profiles at a manageable computational cost, we employed a fragmentation procedure similar to that used in modern parametrization tools.^25^ In this scheme, the three nearest neighbors to the atoms defining the rotatable dihedral are retained explicitly,^11,12^ and all more distant atoms are truncated and capped with hydrogens, except when (1) the bond order to the next atom exceeds 1.75, (2) the next atom is part of a ring, or (3) the atom possesses a Pauling electronegativity greater than 3.0. The molecules and molecular fragments used for the all-atom parametrization are shown in Figure 1.

**Figure 1:**
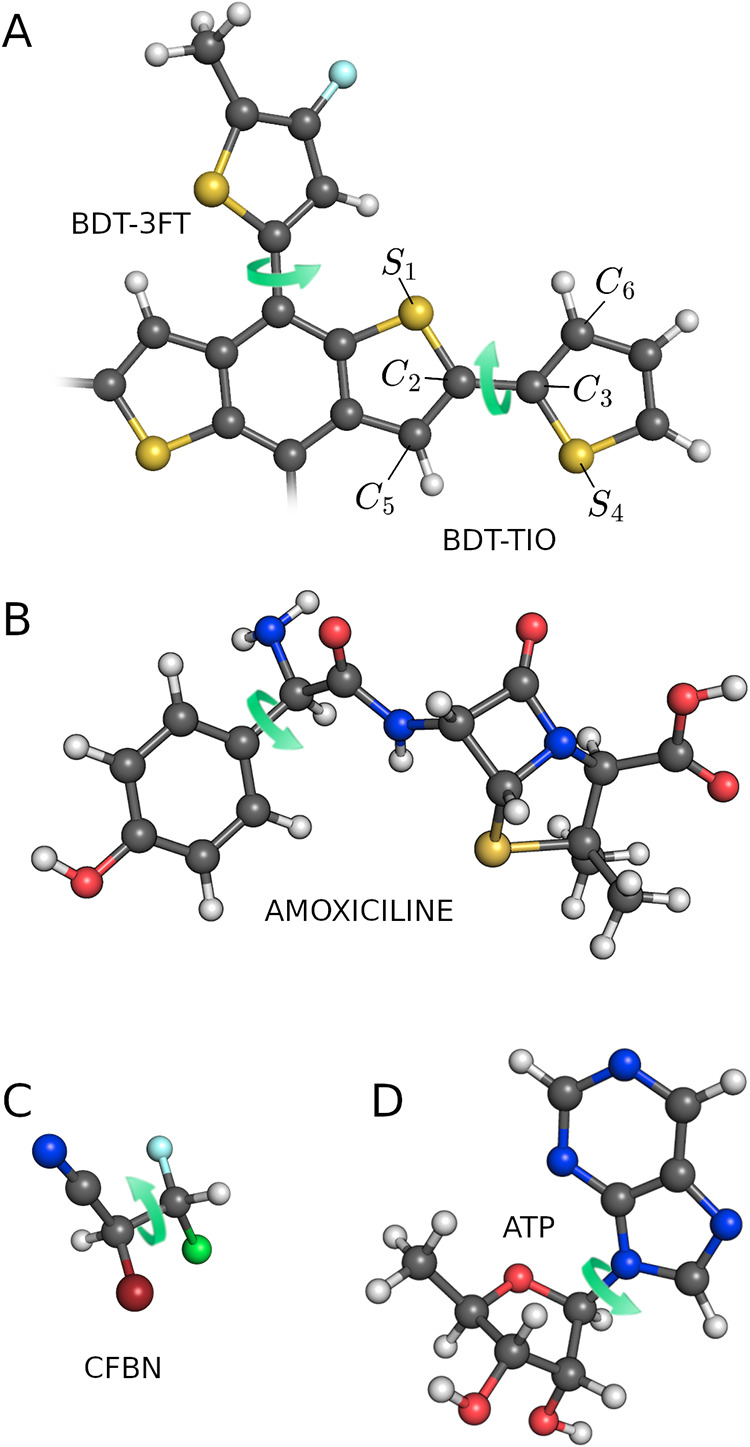
Molecules selected to represent the three torsional categories considered in this study—symmetric, asymmetric, and complex symmetric—for evaluating the FFT parametrization: (A) PBDB-T-2F polymer fragment, displaying symmetric conjugated rotations; (B) Amoxicillin, featuring asymmetric torsions arising from heteroatomic substitutions; (C) (2*S*,3*R*)-2-bromo-3-chloro-3-fluoropropanenitrile, an example of highly asymmetric, halogenated torsions; (D) ATP fragment, characterized by a complex asymmetric multimodal torsion between the ribose sugar and the nitrogenous base or the *N*-glycosidic torsion angle.

#### QM and MM Torsional Profiles

The reference torsional term was computed as

9 $$V_{\mathrm{tors}}\left(\phi \right)=V_{\mathrm{QM}}\left(\phi \right)-V_{\mathrm{MM}}\left(\phi \right)$$

where *V*_QM_ was obtained from relaxed QM scans at 10° increments over 360°, and *V*_MM_ from corresponding MM scans using the same geometries while allowing minimization within the MM force field, turning off the potential energy for that specific dihedral angle. To ensure consistency with the FFT, the final point at 360° is omitted, as it is redundant with the 0° configuration and would otherwise introduce duplication and potential artifacts in the periodic representation.

In our approach, the description of the torsional correction defined in eq 9 is analogous to that found in conventional torsional parametrization methodologies, such as in the work of Hopkins,^14^ Kania,^13^ and Sami,^25^ in which it is defined as the difference between QM and MM energy profiles evaluated along a fixed configuration pathway, assuming that relative energies are meaningful and constant offsets are irrelevant. This interpretation further relies on the MM description of bonded, angular, and nonbonded interactions being considerably accurate, such that the QM-MM difference predominantly reflects torsional contributions.

#### Distribution of Torsional Pathways

For a given dihedral *i*–*j*–*k*–*l*, the torsional contribution must be distributed across all distinct torsional pathways that define that dihedral. If we define *P* as the total number of pathways the energy associated with a single dihedral is therefore

10 $$E_{\mathrm{ijkl}}=\sum_{p}^{P}w_{p}\sum_{n}^{N}V_{\mathrm{ijkl}}\left(n,\phi \right)$$

where the sum over *p* enumerates the different pathways associated with the dihedral, each weighted by a coefficient *w*_*p*_. The innermost sum corresponds to the Fourier terms used to represent the torsional potential. This redundancy of contributing pathways must be taken into account whenever several chemically distinct routes connect the atoms defining a rotatable bond.^47,48^

Accordingly, an appropriate choice of the weights (*w*_*p*_) is required to combine the pathway-specific contributions into a single effective torsional potential. A simple option is to assign equal weights

$$w_{p}=1/P$$

but this implicitly assumes that all pathways contribute equally, which may be an oversimplification. In this work, we consider a constrained least-squares approach, which provides a systematic and data-driven determination of the coefficients. The optimal weights are obtained by minimizing the deviation between the target torsional profile and the weighted combination of pathway-specific contributions. For a set of (*M*) sampled dihedral values, this is expressed as

11 $$\chi^{2}\left(w,\left\{k\right\}\right)=\sum_{k=0}^{M-1}{\left[V_{\mathrm{tors}}\left(\phi_{k}\right)-\sum_{p=1}^{P}w_{p}V_{\mathrm{tors},p}^{\mathrm{FFT}}\left(n,\phi_{k}\right)\right]}^{2}$$

subject to the normalization condition

12 $$\sum_{p=1}^{P}w_{p}=1$$

This constraint ensures that the combined potential preserves the overall energy scale. Additional point-wise weighting factors can be introduced to emphasize low-energy regions, which are more frequently sampled during molecular dynamics simulations. Following Sami and co-workers,^25^ these factors are defined as

13 $$\mu_{k}=\exp\left[-0.2\sqrt{V_{\mathrm{QM}}\left(\phi_{k}\right)}\right]$$

The complete matrix formulation and implementation details are provided in the Supporting Information.

#### Phase Relationship for Torsional Pathways

As illustrated in Figure 1A, the dihedral between two sp^2^ carbons in benzodithiophene and thiophene (BDT-TIO) involves six atoms, generating four valid paths: S_1_–C_2_–C_3_–S_4_, S_1_–C_2_–C_3_–C_6_, C_5_–C_2_–C_3_–S_4_ and C_5_–C_2_–C_3_–C_6_.

In planar systems such as aromatic rings, these paths often exhibit simple phase relationships: some are in phase while others differ by approximately 180°. However, for dihedrals involving mixed hybridizations—particularly when an sp^3^ center is present—the relative phase between paths becomes nontrivial and must be computed explicitly. Figure 2A shows the case of a dihedral formed between an sp^2^ carbon (C_1_) and an sp^3^ carbon (C_2_), where two structural angles, α and β, define the relative phase offset γ between the corresponding torsional paths. In general, the angular difference between dihedral *i*–*j*–*k*–*l* and dihedral *i*_2_–*j*–*k*–*l*_2_ cannot be calculated with a simple difference, but must be determined with an angular difference that accounts for its periodicity. If we define α as the dihedral *i*–*j*–*k*–*l* and β the dihedral *i*_2_–*j*–*k*–*l*_2_, we can compute the angular difference γ with the following equation

14 γ=atan2[sin(β−α),cos(β−α)]

In order to determine an adequate value for the phase offset, we must consider not only one but the whole ensemble of *k*-th QM optimization configurations. For each configuration, we can calculate the angular difference γ and then obtain the average value. Once again we must consider the periodic properties of these angles and obtain the circular mean with

15 $$\mathrm{\gamma ̅}=a\tan2\left(\frac{1}{M}\sum_{k=0}^{M-1}\sin\gamma_{k},\frac{1}{M}\sum_{k=0}^{M-1}\cos\gamma_{k}\right)$$

**Figure 2:**
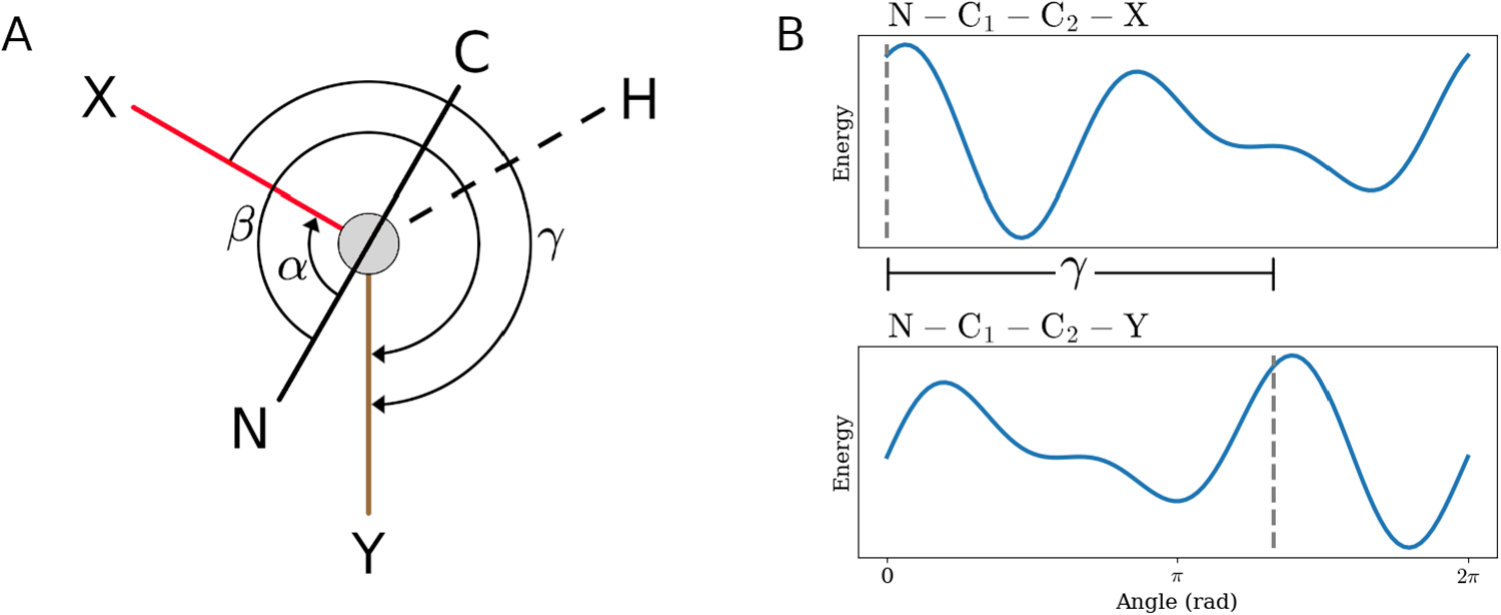
Geometrical definition and phase displacement of distinct torsional paths. (A) Newman projection through bond C_1_–C_2_ of the geometrical construction of the dihedrals N–C_1_–C_2_–X and N–C_1_–C_2_–Y. The local substituent orientations, quantified by the angles α and β, give rise to a phase offset γ between the two torsional pathways. (B) Hypothetical torsional energy curves illustrating how the same underlying torsional potential manifests with a relative phase shift γ when assigned to distinct dihedral paths.

Equations 14 and 15 allow us to avoid discontinuities or jumps when computing the dihedral angles that arise from its periodic nature.^49^ For example, when averaging the angles from the QM configurations for a dihedral close to 180 deg values can be obtain such as 179 or −179. When averaging these values the result is 0, instead of 180.

The resulting phase-shifted torsional energy profiles are illustrated in Figure 2B, demonstrating how the same underlying torsional potential appears displaced when projected onto structurally distinct paths.

The amplitudes *A*_*n*_ associated with the *n*-th frequency remain unchanged for the phase-shifted functions; however, the corresponding phases must be modified. The FFT formalism provides a direct means to calculate the new phases, ν_*n*_, according to the following equations.

16 $$\nu_{n}=\omega_{n}-\frac{2\pi \mathrm{ns}}{M}$$

17 $$s=\frac{\mathrm{\gamma ̅}}{\Delta \phi }$$

where the term γ̅ accounts for the path-dependent phase relationships discussed above, and Δ*ϕ* denotes the spacing between input data points along the ϕ axis. The shift may be applied in either direction by selecting the appropriate sign of γ̅ in eq 17. This procedure yields a complete and internally consistent set of torsional parameters for any dihedral, regardless of symmetry or hybridization.

#### Iterative Refinement of Torsional Potentials

To further improve agreement with the quantum-mechanical target profile, we implemented an iterative refinement procedure following the philosophy of parametrization tools such as Q-Force. At each iteration, the torsional potential is updated according to

18 $$V_{i+1}\left(\phi \right)=V_{i}\left(\phi \right)+\lambda D_{i}\left(\phi \right)$$

where *D*_*i*_(ϕ) = *V*_QM_(ϕ) – *V*_MM,*i*_(ϕ) represents the discrepancy between the QM and MM scans obtained with the current parametrization. The damping factor λ helps for a smoother correction of potential *V*_*i*+1_ and aids to the stability of the algorithm. A value of λ = 0.5 was used. In order to obtain the necessary *A*_*n*_ and ω_*n*_ coefficients for *V*_tors_ in eq 3, FFT was applied on *V*_*i*+1_. This refinement loop gradually drives the torsional profile toward the quantum reference while preserving the frequency content determined by the FFT decomposition.

A key issue arises at this point, which is determining how to truncate the number of terms resulting from applying FFT to the potential *V*_*i*+1_(ϕ), since in principle, as many terms as the Nyquist frequency will be obtained. A first criterion could be to only use terms associated with frequencies less than or equal to *n* = 6. However, even some of these lower-frequency terms may have associated amplitudes of negligible value.

It is impossible to know a priori the set of optimal frequencies that will allow the torsional potential *V*_tors_ to most closely match the *V*_QM_ potential. While using a greater number of terms allows for greater fidelity to the shape, it can also lead to overfitting. Furthermore, considering computational performance, each additional term adds a calculation that reduces that performance. Following the philosophy of spectral analysis,^50^ a methodology has been designed that allows for the selection and testing of different sets of frequencies depending on their contribution weight. These sets of frequencies are applied to the *V*_tors_ potential, and the degree of fit that each allows is determined.

The methodology is summarized as follows: It begins with a number of allowed parameters *F* that determines the number of frequencies to be selected in range from 1,2,...,*N*_max_, where *N*_max_ will be the highest frequency allowed, typically 6. These frequencies will also be the most significant, as determined by their respective amplitudes. For example, for a number *F* = 3, the three most significant frequencies resulting from the FFT decomposition will be selected. These top frequencies will be designated as

19 $$n_{F}=\left\{n_{j},n_{l},...,n_{q}\right\}$$

It should be noted that these selected frequencies are not necessarily consecutive. With this number *F*, iterative refinement will be performed according to eq 18. For each new potential *V*_*i*+1_, *F* frequencies will be selected, and a new MM scan will be performed, the degree of fit of which will be determined using the parameter *R* ^2^. After a number of iterations, the number *F* is incremented by 1, and iterative refinement is performed again with this new criterion to select the most significant frequencies.

After each new MM scan, the resulting *V*_MM_ potential is compared with the target *V*_QM_ potential. If these potentials are sufficiently similar, according to a comparison criterion (e.g., *R* ^2^ greater than 0.98), the procedure is stopped and the set of frequencies *n*_*F*_, as well as their associated values {*A*_*n*_} and {ω_*n*_}, are taken as optimal. If no set of frequencies *F* allows the potential *V*_MM_ to closely resemble the potential *V*_QM_, according to our comparison criterion, then the frequencies and their associated values that came closest are taken.

This procedure is equivalent to an amplitude-based spectral truncation, where the torsional potential is approximated using only the dominant frequency components identified in the Fourier spectrum. In contrast to conventional truncation schemes based on consecutive frequency indices, this approach is less restrictive, as it does not assume that the most relevant contributions necessarily correspond to the lowest frequencies. Moreover is unbiased, since it does not impose a user defined amplitude threshold to consider dominant frequency components, but instead it provides a systematic framework in which the model is constructed incrementally testing all the frequencies subsets. Such an approach ensures that the resulting model captures the essential features of the energy landscape while maintaining a compact representation.^51^ The flowchart for this procedure is schematized in Figure 3.

**Figure 3:**
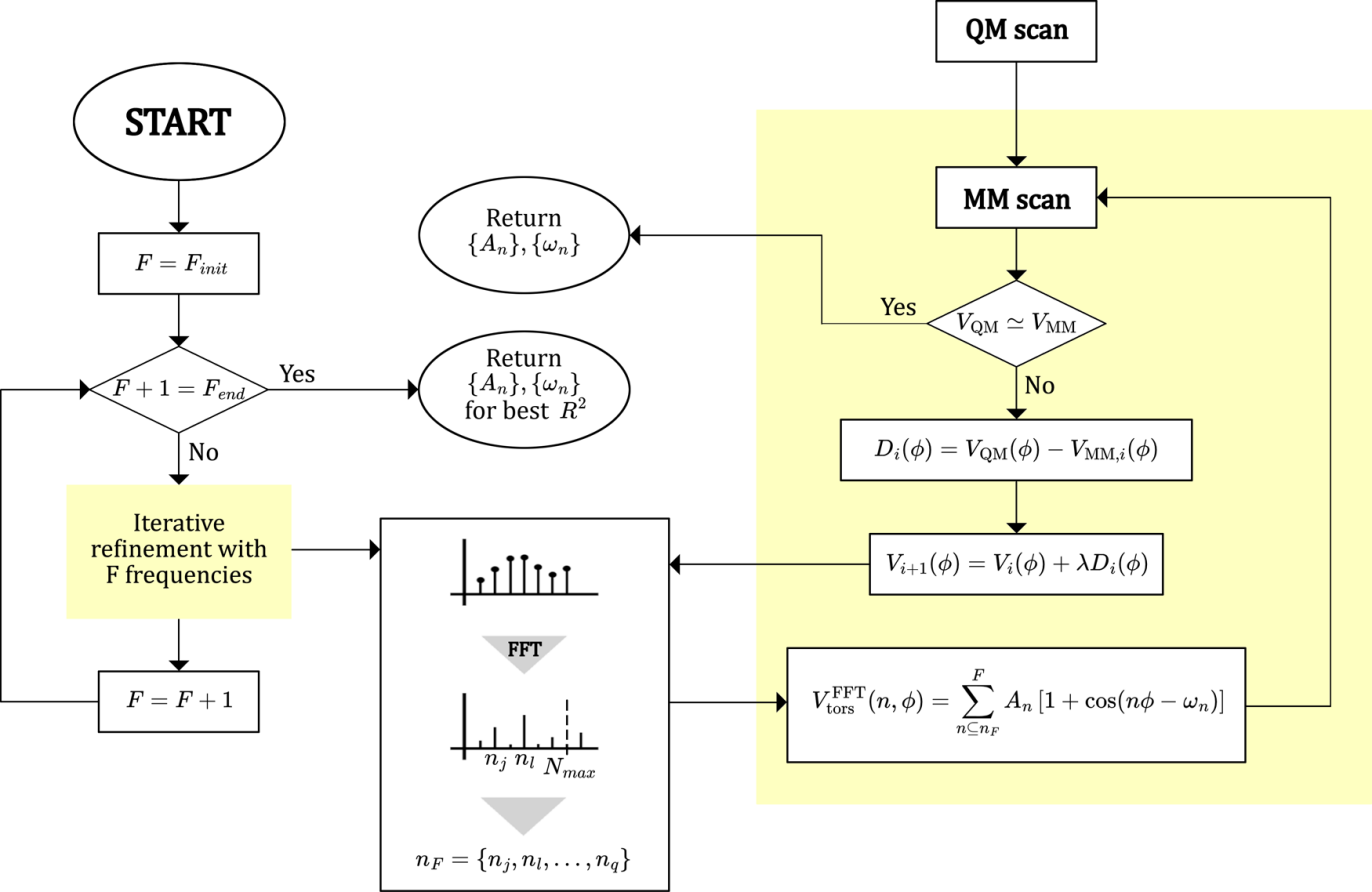
Flowchart of the iterative spectral refinement procedure used to determine torsional potentials. The algorithm consists of two nested loops. In the inner loop, the difference between QM and MM energy profiles is iteratively corrected using a truncated Fourier representation obtained from FFT. In the outer loop, the number F of Fourier terms is progressively increased until the desired agreement between QM and MM energy profiles is achieved.

### Coarse-Grained Torsional Parametrization Using FFT

The FFT-based torsional parametrization introduced here is fully general and independent of the underlying CG force field. Its implementation does not rely on any feature unique to Martini, and the workflow can be transferred directly to any CG representation. To illustrate the method and probe its performance across contrasting chemical contexts, we selected two systems with existing Martini 3 mappings (Figure 4). (1) The first system, the molecular photoswitch MS–Z, whose CG model was developed by Marrink and collaborators,^52^ exhibits symmetric and highly regular torsional profiles, making it an ideal controlled benchmark for FFT. The corresponding atomistic model was constructed with Q-force in conjunction with Gaussian 16. (2) The second system, the intrinsically disordered peptide Aβ42, represents the opposite extreme: its CG backbone dihedrals are multimodal, heterogeneous, and strongly asymmetric. Accurate torsional potentials are essential to prevent unphysical collapse,^53^ and previous parametrizations relied on manual adjustment, underscoring the need for an automated strategy.^53^ The atomistic model was built with CHARMM-GUI and simulated with the CHARMM36m force field. Four representative backbone dihedrals were selected to span the diversity of PES shapes characteristic of disordered proteins.

**Figure 4:**
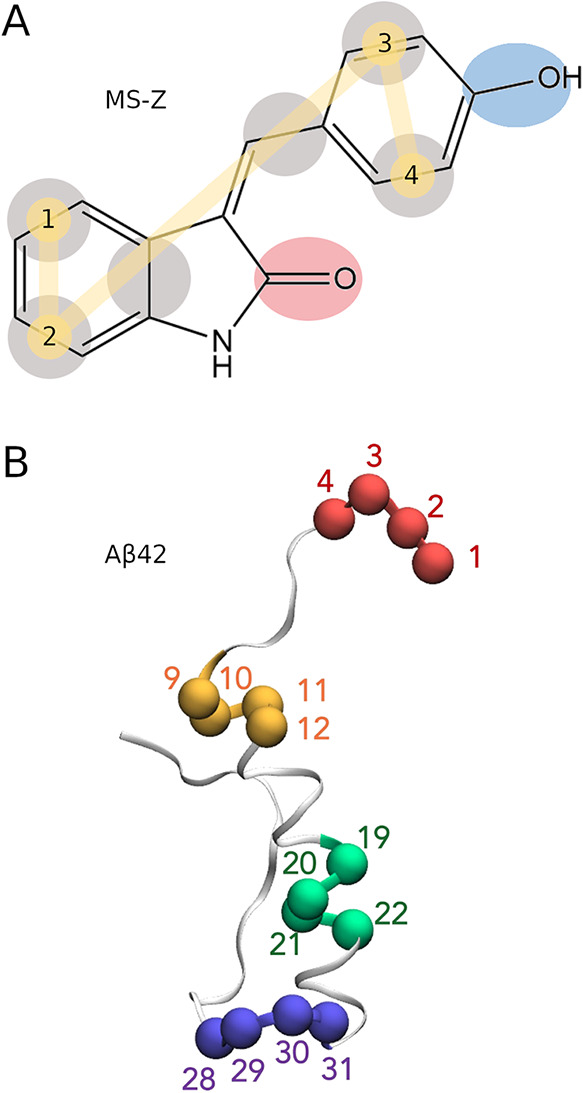
Selected coarse-grained systems used to evaluate the FFT-based torsional parametrization: (A) MS–Z molecular photoswitch; (B) Aβ42 peptide, with backbone bead quadruplets corresponding to the analyzed CG dihedrals highlighted.

#### Bottom-Up Strategy

The parametrization of CG torsions followed a bottom-up scheme in which atomistic simulations are used as the reference for the CG model. The atomistic trajectories are mapped onto the CG bead representation, and the resulting distributions are used to derive bonded potentials through Boltzmann Inversion. For each system, a 1 μs atomistic MD simulation in water was performed, from which the distributions of the selected dihedral angles were extracted using the MDAnalysis^54,55^ Python library. Torsional energy profiles were obtained by Boltzmann inversion of the angular distributions according to

$$E_{k}=-k_{B}T\ln\left(\frac{a_{k}}{A}\right)$$ 20

where *E_k_* is the energy associated with the dihedral angle ϕ_*k*_, *a*_*k*_ is the number of occurrenes, *A* = ∑_*k*_ *a*_*k*_, *k*_B_ is the Boltzmann constant, and *T* is the temperature. We can obtain the reference torsion potential with

$$V_{\mathrm{tors}}\left(\phi \right)=E_{\mathrm{AA}}\left(\phi \right)-E_{\mathrm{CG}}\left(\phi \right)$$ 21

the torsional profile was then decomposed using the FFT according to eq 8 to obtain the coefficients of the functional form in eq 1. All distributions for dihedral angles were obtained in the range of 0 to 360 degrees using a bin size of 1.

#### Iterative CG Refinement

Starting from these initial FFT-derived potentials, CG simulations of 500 ns were carried out for each molecule. From the resulting trajectories, the dihedral distributions and corresponding Boltzmann-inverted energy profiles were recomputed. At this stage, two torsional profiles are available for each angle: one from the AA reference and one from the current CG iteration. To compare them on equal footing, both profiles were smoothed with a FFT representation allowing up to 20 frequency components, and their difference was used to update the torsional potential. The iterative scheme follows

22 $$V_{i+1}\left(\phi \right)=V_{i}\left(\phi \right)+\lambda \left[E_{\mathrm{AA}}\left(\phi \right)-E_{\mathrm{CG},i}\left(\phi \right)\right]$$

where *E*_AA_(ϕ) is the target AA energy profile and *E*_CG,*i*_(ϕ) is the *i*-th CG energy profile. This expression is the direct analogue of the IBI procedure commonly used to derive potential functions.^56,57^ As in the AA section, the iterative correction enables systematic refinement of the torsional term until the CG and AA profiles converge. Again a value of λ = 0.5 was used.

The frequency selection procedure described in Section AA is too expensive to apply in CG models, as it would require a molecular dynamics simulation at each iteration of the fitting process. Given the lower resolution of CG representations, we use a simplified frequency selection strategy. We select Fourier components based on their relative amplitudes. Specifically, only frequencies whose amplitudes exceed a fraction κ of the maximum amplitude are retained

23 $$n_{T}=\left\{n|A_{n}\geq \kappa A_{\max}\right\}$$

Typical values of κ range between 0.05 and 0.2. This criterion removes negligible contributions while limiting the number of retained terms, thereby reducing the risk of overfitting. The choice of κ introduces a degree of user-defined bias. However, components with amplitudes above approximately 10% of the maximum capture the dominant features of the torsional profile. In this work, we use κ = 0.08 for frequency selection in both AA and CG energy curves, with a maximum frequency of *n* = 20. For the final potential fitting, we use κ = 0.1 together with a maximum frequency of *n* = 6, further reducing the number of terms and avoiding overfitting.

As a final validation step, the torsional potentials derived using the present methodology were compared against those obtained from Swarm-CG and the Martini 3-IDP force field. In the first case, the molecule MS-Z was parametrized using Swarm-CG. For comparison with Martini 3-IDP, the intrinsically disordered peptide Histatin 5 was selected as a representative test system, given its well-known conformational flexibility and relevance as a benchmark for coarse-grained models of disordered proteins. The comparison focuses on the effective torsional behavior sampled during simulations, allowing assessment of whether the proposed parametrization reproduces the conformational preferences captured by an established coarse-grained framework.

## Results

### Validation of the FFT-Based Torsional Parametrization in All-Atom Models

#### BDT-TIO Fragment: Symmetric Multimodal Torsions

A fragment of the PBDB-T-2F donor polymer, widely used in organic photovoltaic applications, was selected to evaluate torsions between a benzodithiophene (BDT) core and its adjacent thiophene (TIO) or 3-fluorothiophene (3FT) rings. These dihedrals are expected to be symmetric and therefore provide an ideal benchmark for assessing the ability of the FFT approach to reproduce smooth, multimodal torsional energy profiles. Quantum-mechanical scans were obtained at the ωB97XD/6–31G­(d,p) level, consistent with ref 58, and the corresponding MM scans were performed using OPLS parameters generated with Q-force. For this case only, due to its simple symmetry, the scans were performed with 15° increments.

As shown in Figure 5, the BDT-TIO dihedral exhibits symmetric QM and MM potential-energy curves, and their difference—corresponding to *V*_tors_—likewise displays a symmetric multimodal form. For this first case, we compare the performance of the RB-type potential with that of the FFT-derived potential in fitting this class of torsional profile. It is worth noting that the RB functional form cannot represent profiles requiring cosine components with frequencies higher than five, as can be shown by expanding eq 2 in terms of its cos­(nϕ) components. To ensure a fair comparison, the FFT-derived potential was therefore restricted to a maximum frequency of five (FFT5). Both the RB fit and the FFT5 torsional potential reproduce the reference profile with high accuracy. These results indicate that, for symmetric multimodal torsions of this type, the FFT parametrization performs equivalently to the RB formulation and does not present any intrinsic limitations.

**Figure 5:**
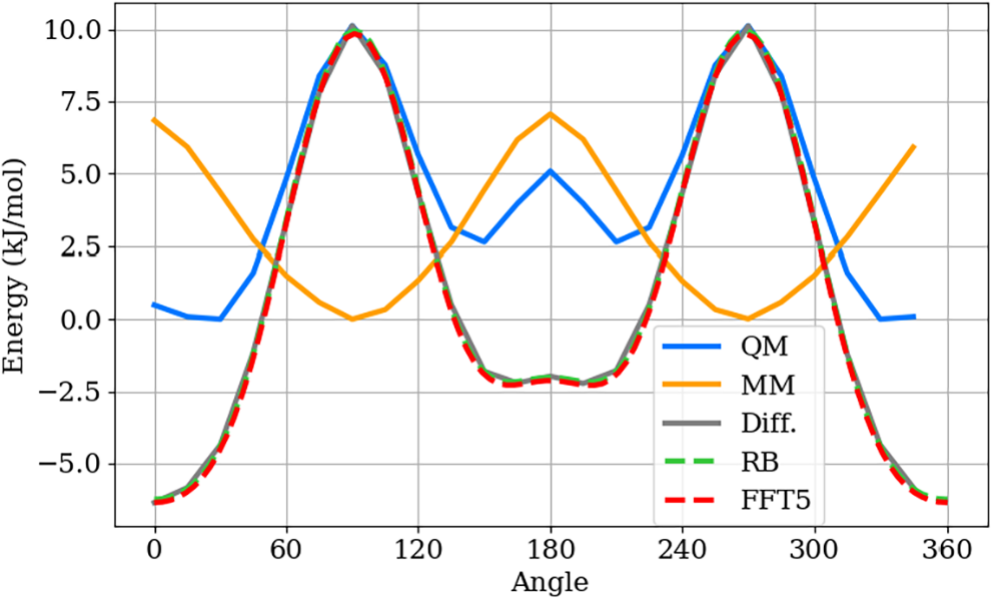
Torsional energy profiles for the BDT–TIO dihedral in the PBDB-T-2F fragment. Both QM and MM scans exhibit a symmetric multimodal shape, and the resulting torsional correction term *V*_tors_ = *V*_QM_ – *V*_MM_ is likewise symmetric. Fits obtained using the RB polynomial and the FFT-based approach both reproduce the target profile with high accuracy.

#### Amoxicillin Isomer: Asymmetric Torsion

The amoxicillin isomer previously analyzed by Hopkins^14^ contains an asymmetric dihedral located adjacent to a chiral center. Because the QM–MM energy differences for this torsion were explicitly reported in ref 14, these values were used directly as a benchmark for evaluating the FFT-based parametrization. This allows for an exact comparison with the four-term truncated Fourier potential obtained via LLS in that work.

For this asymmetric profile, shown in Figure 6, the LLS fit using a maximum frequency of 4 (LLS4) and the four-term FFT fit (FFT4) both resulted in near perfect fit, with the same *R* ^2^. This indicates that the LLS and FFT provide the same coefficients. However, FFT provides a key practical advantage: The method naturally generates all frequency components from the discrete data up to the Nyquist frequency, thereby allowing the user to freely select the number of terms to retain. In contrast, LLS requires the number of cosine terms to be fixed a priori. This advantage becomes particularly valuable when determining how to truncate the frequency components.

**Figure 6:**
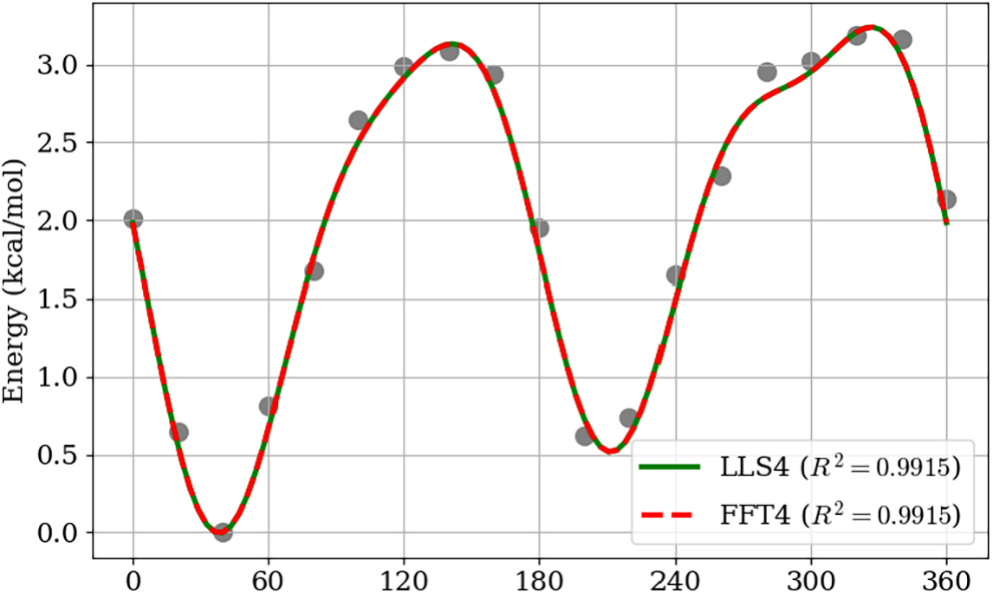
Comparison between the four-term LLS fit (LLS4) and the four-term FFT fit (FFT4) for the asymmetric torsional profile of the amoxicillin isomer.

#### Designed CFBN: Strongly Asymmetric Torsion

To probe extreme cases of torsional asymmetry, we designed the molecule (2*S*,3*R*)-2-bromo-3-chloro-3-fluoropropanenitrile (CFBN), which contains a rotatable dihedral connecting two chiral carbons. Highly electronegative substituents were selected to suppress conformational rearrangements along the PES and ensure continuous scans. QM scans were performed using PBEPBE/6–31+G­(d), and the corresponding MM scans employed OPLS parameters generated with Q-force.

As shown in Figure 7, both the QM and MM torsional profiles display the expected asymmetric shape due to the chirality of the atoms involved. To directly compare the performance of FFT against the RB potential, again the FFT fit was restricted to a maximum frequency of 5 (FFT5). The RB potential is unable to reproduce the asymmetry of the QM–MM difference profile, whereas the FFT5 fit captures the overall shape with an *R* ^2^ of 0.94. Allowing higher frequencies in the FFT representation naturally improves the agreement with the target PES. In this case, comparison with the LLS method is unnecessary. As demonstrated for the amoxicillin torsion, FFT produces coefficients essentially identical to those obtained by LLS when fitted with the same number of terms, but without requiring any modification of the fitting strategy. FFT, therefore, provides a more general and flexible framework for reproducing asymmetric torsional potentials.

**Figure 7:**
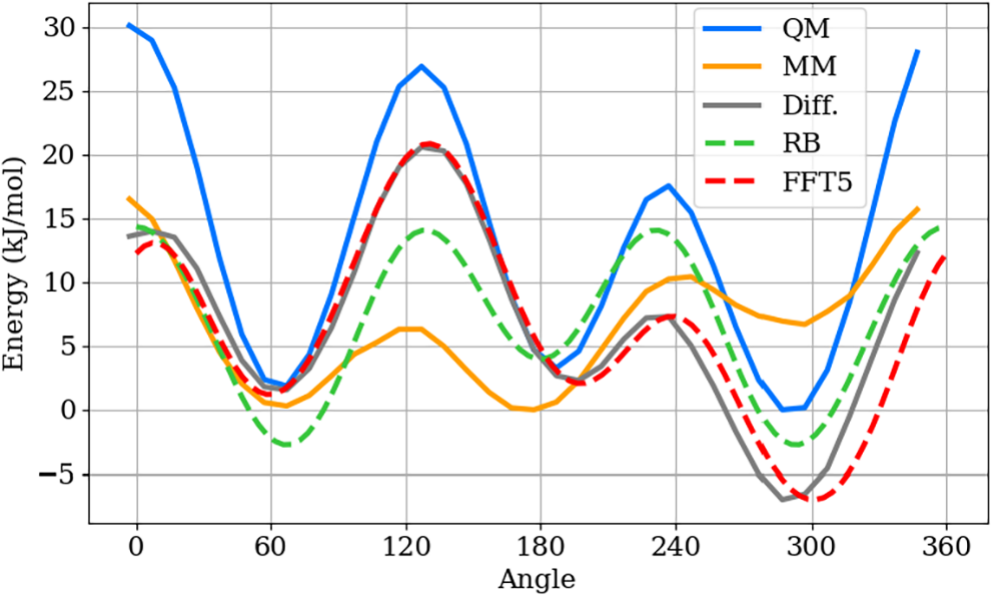
Torsional energy profiles for the rotatable dihedral in CFBN. The QM and MM scans both exhibit strongly asymmetric shapes arising from the two chiral carbons, making this system a crucial test of the FFT torsional parametrization.

#### BDT-3FT Fragment: Complex Symmetric Torsion

Figure 8 shows that significant discrepancies between the QM and MM scans around 180° produce a broad, flattened minimum whose shape is difficult to reproduce, even though the underlying torsion is symmetric. Although such cases may be uncommon, they are essential for assessing the limitations of any fitting methodology. As previously discussed, the RB functional form cannot represent profiles requiring cosine components with frequencies higher than five. The shape of the BDT–3FT torsion clearly demands higher-frequency modes, and this becomes evident when comparing the fits: allowing FFT to include up to ten frequency components (FFT10) substantially improves the agreement with the QM–MM difference curve, particularly in the 160°–200° region. Nevertheless, even FFT10 retains a residual deviation of approximately 2.5 kJ/mol, which is markedly smaller than the nearly 10 kJ/mol discrepancy obtained with the RB potential. QM treatment was the same as in BDT-TIO, MM scans were generated using GAFF2 force field.

**Figure 8:**
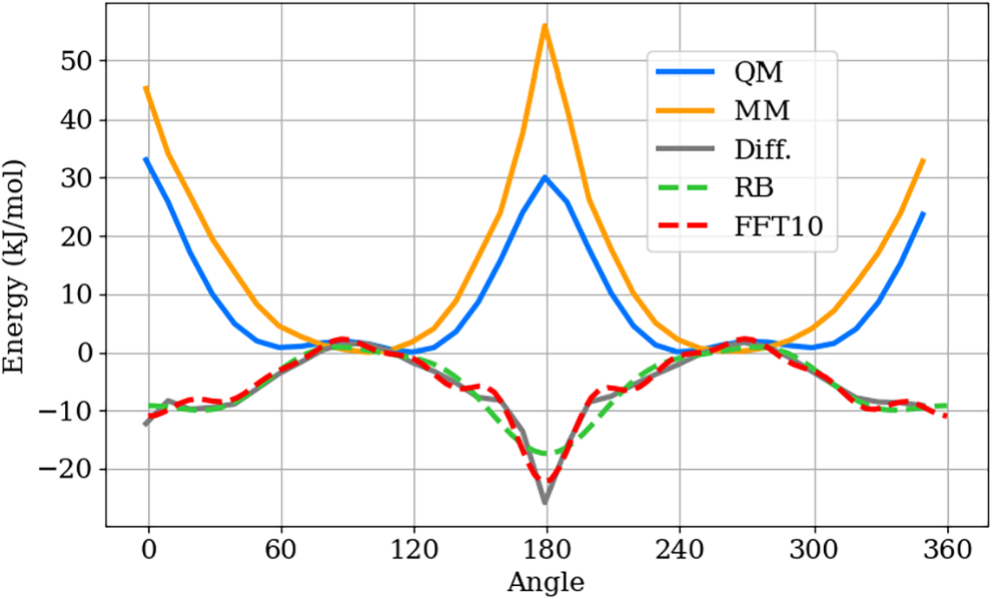
Torsional energy profiles for the rotatable dihedral in BDT-3FT. The difference between the QM and MM scans produces a narrow minimum around 180, creating a challenging target for parametrization due to the steep curvature and highly localized nature of the well.

It is important to note that although this extreme case challenges both RB and FFT, the iterative refinement procedure introduced in eq 18 can further reduce the residual error, even when starting from an RB-type potential.

#### ATP Fragment: Multi-Path and Phase-Shift Complexity

A fragment of ATP containing only the ribose and nucleobase was examined to assess FFT performance in cases where multiple distinct torsional pathways contribute to a single torsion. The dihedral connecting these units is asymmetric and requires the phase-offset correction described earlier (Figure 2). QM scans were performed at the PM6 level to avoid discontinuities caused by large structural rearrangements observed at higher levels of theory. In some systems, torsional rotation may be strongly coupled to structural rearrangements, particularly at the DFT level, leading to multidimensional energy landscapes that cannot be reduced to a one-dimensional torsional profile. In such cases, semiempirical methods (e.g., PM6) can provide a practical approximation by smoothing the potential energy surface and enabling the extraction of an effective torsional potential. This approach assumes that the torsional degree of freedom remains dominant; when strong coupling persists, a one-dimensional representation may no longer be appropriate. MM scans were performed using OPLS parameters generated with Q-force. The torsional PES of this fragment exhibits significant conformational changes during the scan, leading to discontinuous energy jumps and preventing the smooth profiles obtained for previous systems. One such rearrangement occurs when the dihedral C_1_–N_2_–C_3_–O_4_ rotates from 300° to 310°, during which the methyl group switches from an equatorial to an axial orientation (Figure 9A). As expected, the torsion is asymmetric due to the chirality at *C*_3_, a feature confirmed by the QM and MM scans shown in Figure 9C. To parametrize this torsion, the FFT-based approach was applied using a maximum frequency of 6. To evaluate the effect of distributing the torsional potential along the dihedral pathways, two strategies were examined: (i) applying the full torsional potential to a single path (1-P), C_1_–N_2_–C_3_–O_4_, and (ii) distributing it across the four relevant paths (4-P): C_1_–N_2_–C_3_–O_4_, C_1_–N_2_–C_3_–C_6_, C_5_–N_2_–C_3_–O_4_, and C_5_–N_2_–C_3_–C_6_.

**Figure 9:**
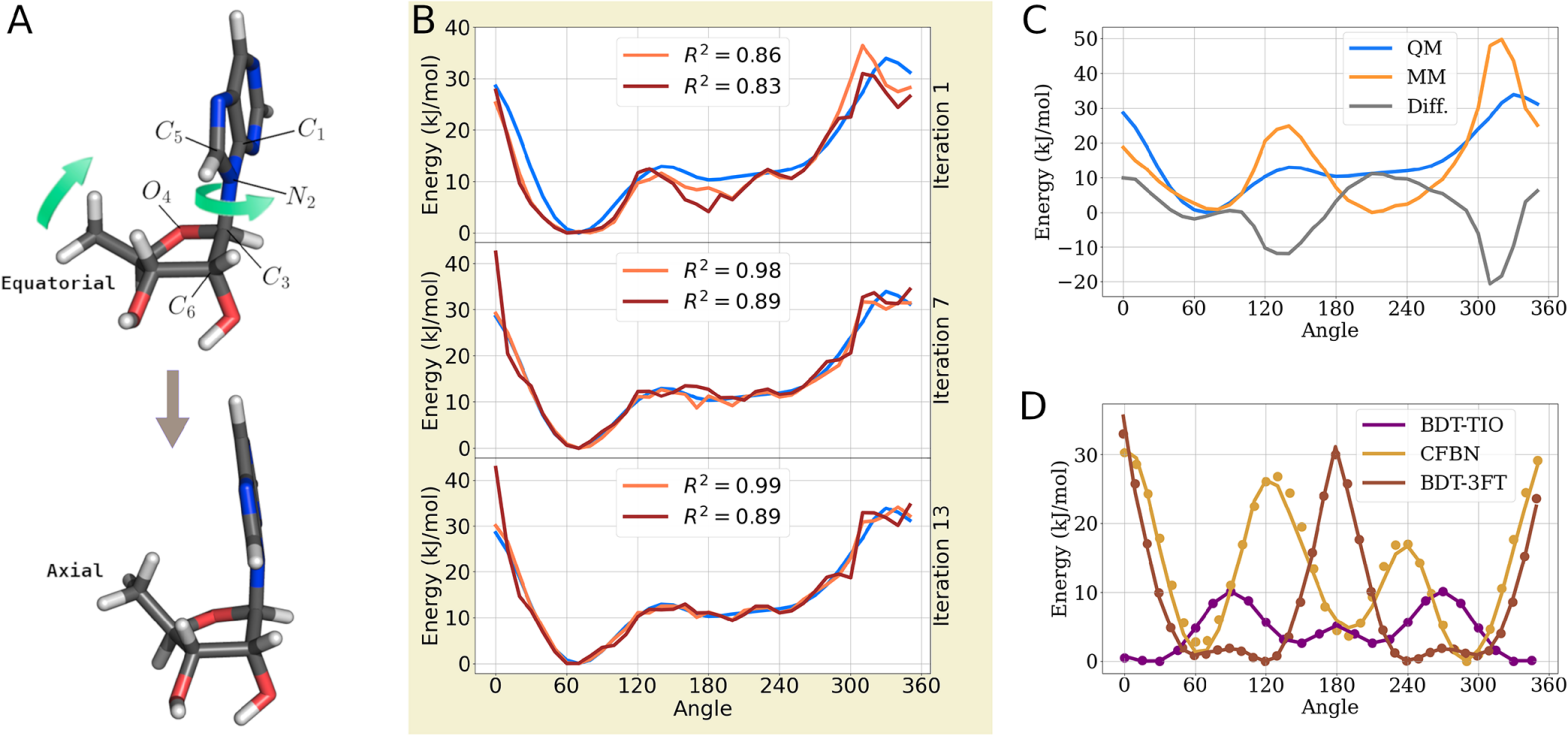
(A) Conformational change observed in the ATP fragment during the torsional scan. (B) Evolution of the MM torsional profiles during the iterative refinement procedure when the torsional potential is applied to a single path (1-P, crimson) or distributed over four paths (4-P, coral). (C) Initial QM and MM scans for the *C*_1_–*N*_2_–*C*_3_–*O*_4_ dihedral. (D) Final dihedral fits for the BDT–TIO (symmetric), BDT–3FT (complex symmetric), and CFBN (highly asymmetric) torsions. QM scans are shown as points and the converged MM profiles as lines. After iterative refinement, the FFT-derived torsional potentials reproduce the QM targets with *R* ^2^ > 0.98 in all three systems, including the challenging BDT–3FT and CFBN cases.

Figure 9B shows the iterative refinement results. Already in the first iteration, the 4-P model produces a markedly better MM torsional profile than the 1-P model. In both cases, the largest discrepancies occur between 300°–360°, corresponding to the conformational transition described above. By the seventh iteration, the 4-P model achieves *R* ^2^ = 0.98, whereas the 1-P model remains at *R* ^2^ = 0.89 and substantially overestimates the QM barrier at 0°. After 13 iterations, the 4-P model reaches an essentially perfect fit, while the 1-P model shows no further improvement.

These results demonstrate that correctly distributing the total torsional potential *V*_tors_ across all contributing paths is crucial for accurate parametrization. The phase-shift correction introduced in this work in eq 16 was also effective in assigning the appropriate phase ν_*n*_ for each path.

#### FFT-Iterative Spectral Refinement

The combined use of FFT spectral selection and iterative refinement according to eq 18 yielded MM torsional profiles for the CFBN, BDT–TIO and the BDT–3FT systems that reproduce the QM reference surfaces with *R* ^2^ > 0.98, using different sets of frequencies (Figure 9D). For these systems a maximum frequency of 6 was allowed, values of *F*_init_ = 1 and *F*_end_ = 6 were used, and the permitted number of refinement iterations was set to 20. These parameters were set as default for the program dihefit_fft.py, but they can be modified by the user. Table 1 summarizes the Fourier components selected for each molecule as a function of the target fitting accuracy, expressed in terms of the *R* ^2^ score. For all systems, the parametrization procedure identifies a small subset of dominant frequencies that progressively increases as higher accuracy thresholds are imposed. A clear trend is observed in all cases: lower accuracy thresholds (*R* ^2^ ≥ 0.96) can be achieved using a limited number of low-order Fourier components, typically corresponding to the fundamental frequencies (*n* = 1–3), although in the more complex cases such as BDT-3FT and ATP higher frequencies are necessary even at this threshold. As the required accuracy increases, additional higher-order terms are incorporated, reflecting finer features of the torsional energy landscape. Notably, BDT-TIO reaches *R* ^2^ = 0.98 using only half the number of Fourier terms required for the best-fit model, and without requiring the *n* = 3 component. For the case of BDT-3FT, it does not require the *n* = 4 Fourier component to achieve its best fit, although it is not able to reach the (*R* ^2^ ≥ 0.99) threshold. The ATP system exhibits the largest number of required components, particularly at higher accuracy thresholds, suggesting a more complex torsional energy profile with significant contributions from multiple frequencies. This is consistent with the increased conformational coupling and structural complexity discussed previously.

**Table 1:** Final Sets of Frequencies Obtained for Each Molecule within a Given *R* ^2^ Score

	scores			
dihedral	$R^{2}\geq 0.96$	$R^{2}\geq 0.98$	$R^{2}\geq 0.99$	best *R* ^2^
CFBN	1,2,3	1,2,3,4	1,2,3,4,6	1,2,3,4,5,6
BDT-TIO	1,2,4	1,2,4	1,2,3,4	1,2,3,4,5,6
BDT-3FT	1,2,3,5	1,2,3,5,6		1,2,3,5,6
ATP	1,2,3,4	1,2,3,4,5,6	1,2,3,4,5,6	1,2,3,4,5,6

Overall, the method provides a systematic and physically interpretable way to balance model accuracy with the minimization of the number of Fourier terms in the parametrization of torsional potentials.

### Validation of the FFT-Based Torsional Parametrization in Coarse-Grained Models

#### MS–Z: Symmetric Multimodal Torsions

The torsion selected from the MS–Z photoswitch exhibits a symmetric, multimodal distribution in the AA trajectory, with four dominant peaks near 60°, 120°, 240°, and 300° (red shading in Figure 10A). Boltzmann inversion of this distribution obtained with eq 20 yields a symmetric torsional energy profile with barrier heights of approximately 6 kJ/mol (blue shading in Figure 10B), which is accurately reproduced by the initial FFT-derived energy fit (blue solid line). At iteration 0, the torsional potential is off (black dashed line in Figure 10B), resulting in broad sampling of the CG torsional distribution (gray shading in Figure 10A) that does not match the target AA distribution. The corresponding CG energy profile (orange shading in Figure 10B) reflects this discrepancy relative to the AA reference. The FFT-derived fit generates a smooth representation of the CG energy profile (solid orange line). The difference between AA energy and the CG energy is obtained by directly subtracting the CG-fit from the AA-fit for each iteration (solid black line). Only for iteration 0 the new potential (brown dashed line) is generated directly from the AA-energy, but in advance the new potential is generated by adding this difference to the current torsional potential, according to eq 22. The coefficients of this updated potential are then determined using the FFT formalism. A new CG simulation is subsequently performed using this updated potential.

**Figure 10:**
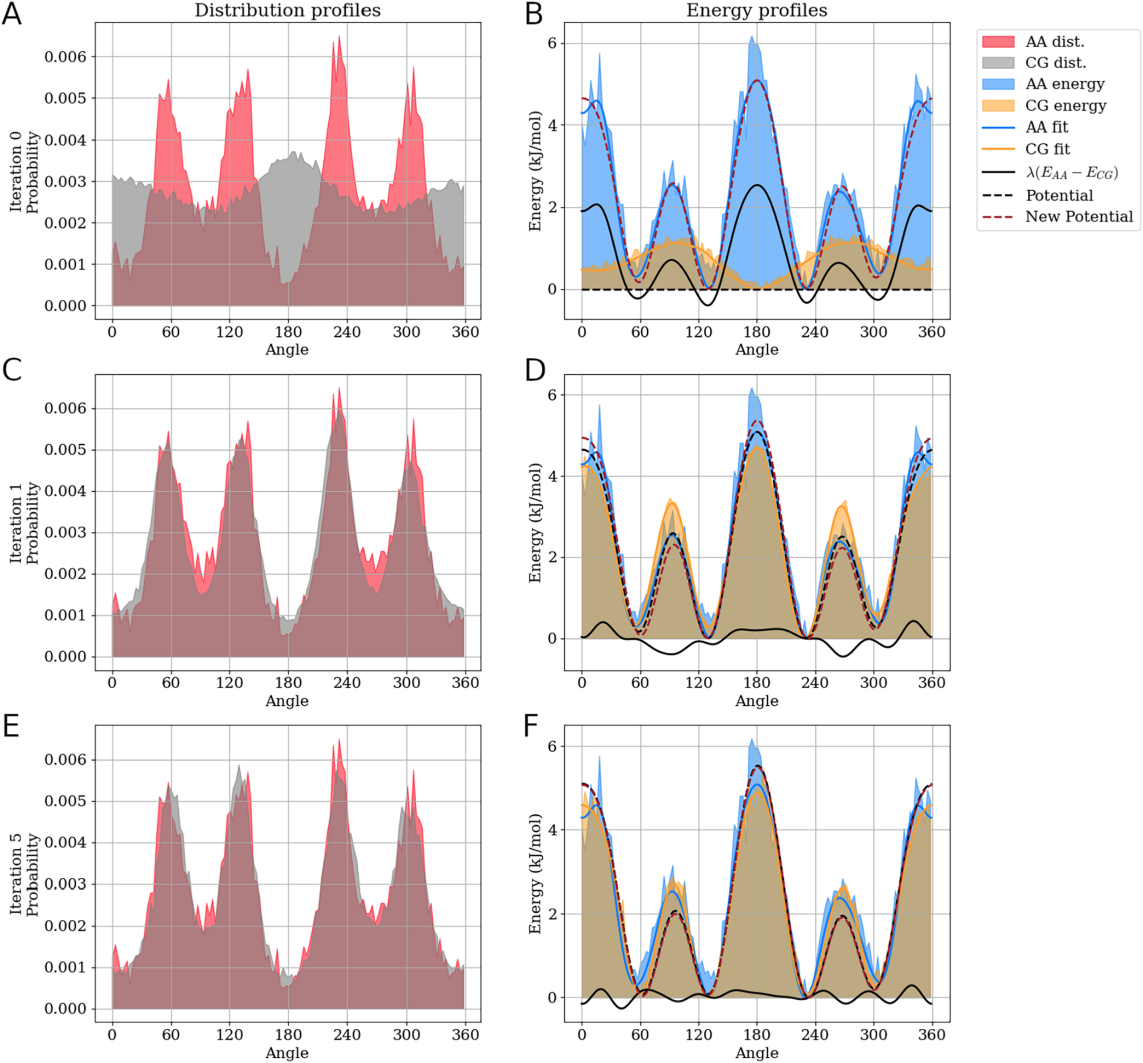
Iterative FFT + IBI refinement of the MS–Z coarse-grained torsion. (A,C,E) Dihedral distributions at iterations 0, 1, and 5 showing the AA target (red) and current CG (gray). (B,D,F) Corresponding AA Boltzmann-inverted reference (blue), the CG energy (orange), the current dihedral potential for CG model (dashed black), the AA–CG difference (solid black), and the updated FFT-derived potential (dashed brown). The CG model systematically approaches the AA target, with both the energy landscape and probability distribution converging to near-perfect agreement as the potential is refined.

After the first iteration (Figure 10C–D), the CG and AA torsional distributions show substantially improved agreement, and the required energetic correction decreases to roughly 1 kJ/mol. However, the CG energy profile still underestimates the correct barrier height at 0°,180° and 360°, while overestimates it at 90° and 270°. To correct these finer discrepancies a correction of slightly more than 0.5 kJ/mol is applied, producing a further updated torsional potential (brown dashed line), which is higher at 0°,180° and 360°, and lower at 90° and 270°. The IBI procedure is repeated under this same scheme, and by the fifth iteration (Figure 10E–F), the AA and CG torsional distributions exhibit an almost perfect overlap, indicating that the FFT-derived torsional potential has effectively converged.

Table 2 lists the final FFT coefficients. Frequencies 2, 4, 5, and 6 contribute to the optimized potential, with the *n* = 6 term retaining a non-negligible amplitude (0.24 kJ/mol). This contribution represents approximately 10% of the dominant *n* = 2 component. Importantly, this demonstrates that—even for a torsion that is formally symmetric—the potential contains high-frequency components that cannot be captured by the Ryckaert–Bellemans functional form, which is restricted to cosine orders up to 5.

**Table 2:** Final FFT Coefficients for the CG Torsional Potential of MS–Z

frequency	amplitude (kJ/mol)	phase (deg)
2	2.22	0
4	1.44	0
5	0.45	182
6	0.24	198

#### Aβ42 Peptide: Asymmetric and Highly Heterogeneous CG Torsions

Figure 11 shows the AA and CG torsional distributions for the four selected backbone dihedrals of Aβ42. As expected for an intrinsically disordered protein (IDP), all AA distributions (red histograms) are multimodal and asymmetric, providing a stringent test of the FFT approach. For the first three dihedrals (residues 1–2–3–4, 9–10–11–12, and 19–20–21–22), the AA profiles exhibit a well-defined peak near 60°, consistent with the transient helical structure. Smaller peaks also appear at distinct locations: the 9–10–11–12 torsion displays local maxima near 100°, 150°, and a more pronounced one around 270°, reflecting the highly flexible behavior of this N-terminal region. The 28–29–30–31 dihedral, located near the C-terminus, presents the broadest and most irregular distribution, as expected for the disordered tail. After applying FFT to the AA-derived profiles and refining the CG potentials through ten IBI iterations, the resulting potentials (black dashed lines) successfully reproduce the overall shapes of the target AA distributions. In the CG simulations (gray histograms), most secondary peaks are captured with excellent accuracy, including the 100° and 150° features of the 9–10–11–12 torsion. This demonstrates that even with a conservative frequency cutoff (*n*_max_ = 6), FFT is capable of capturing subtle energetic modulations.

**Figure 11:**
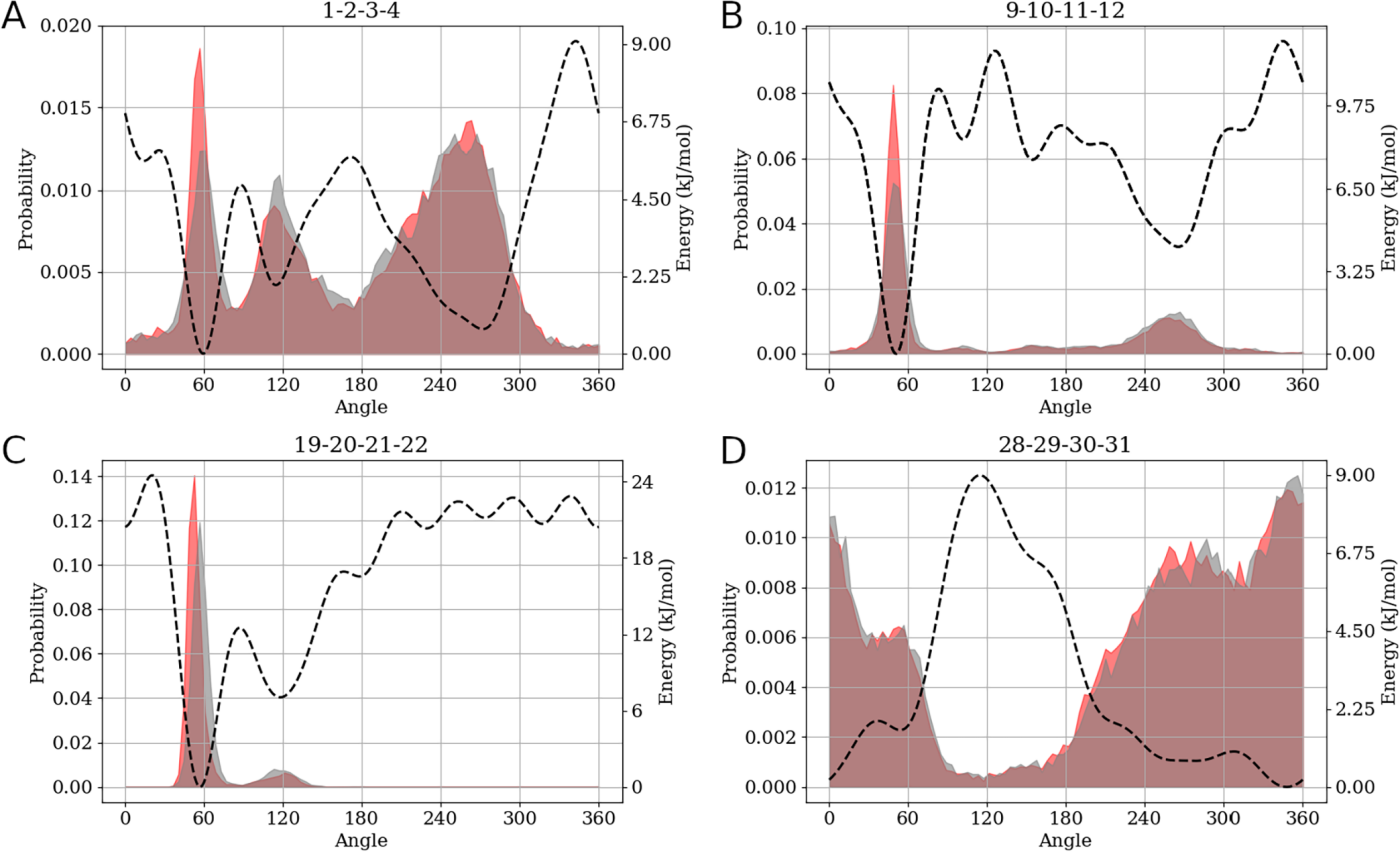
Torsional distributions and final FFT-refined potentials for the four selected backbone dihedrals of the Aβ42 coarse-grained model. Panels (A–D) correspond to dihedrals 1–2–3–4, 9–10–11–12, 19–20–21–22, and 28–29–30–31, respectively. For each dihedral, the all-atom (AA) distribution obtained from the atomistic trajectory is shown in red, and the corresponding coarse-grained (CG) distribution after iterative refinement is shown in gray. The final FFT-derived torsional potential, obtained after 10 IBI iterations, is shown as a black dashed curve (right axis). Together, the panels illustrate how the FFT+IBI procedure captures both the highly asymmetric and multimodal features characteristic of IDP backbone dihedrals in Martini 3-IDP.

A limitation arises for the narrow 60° peak in the first three torsions: although the FFT potential positions its deepest minimum correctly at 60°, its curvature is insufficiently steep to fully recover the sharpness of the AA distribution. Higher-frequency components would be required to describe such a narrow well with greater precision. For the remaining sectors of the profile, the CG distributions achieve excellent agreement.

It is also important to mention the scope of the present coarse-grained analysis. In Martini and related CG models, backbone torsions often require additional correction terms to recover the structural preferences observed at the atomistic level. In this work, however, our goal is not to evaluate the performance of Martini for intrinsically disordered proteins, nor to compare it against existing structural–restraint schemes. Instead, the objective is strictly methodological: to demonstrate that FFT, combined with IBI refinement, provides a simple and efficient route for matching the coarse-grained torsional distributions to their all-atom counterparts. The examples chosen here (MS–Z and Aβ42) therefore serve only as test systems to validate the generality and robustness of the parametrization procedure, rather than as case studies of CG model accuracy.

#### Comparison with Existing Methodologies

For the MS-Z system, Figure SF1 in the Supporting Information compares the target torsional probability distribution (AA, red) with those obtained from the proposed FFT-based parametrization (gray) and the Swarm-CG method (yellow). Overall, the FFT-based model reproduces the main features of the reference distribution with good fidelity, capturing both the positions and relative intensities of the dominant peaks across the full angular range. Swarm-CG also captures the overall periodicity of the distribution; however, differences are observed in several regions, particularly in peak heights and widths. These deviations indicate differences in how the underlying torsional landscape is represented by the two approaches. It should be noted that minor modifications to the original MS-Z CG model were required to enable parametrization with Swarm-CG (limited to the definition of virtual sites and constraints), which do not affect the dihedral definition. The number of terms in the potential was also set to (*n* = 6).

Figure SF2 in the Supporting Information shows the dihedral angle distributions obtained from coarse-grained simulations using the present parametrization method and the Martini 3-IDP model, in comparison with the reference AA data for Histatin 5. The distributions obtained with the proposed model closely reproduce the key features of the AA reference, including the positions and relative populations of the dominant conformational states. The Martini 3-IDP model also captures the general features of the distribution, although some differences are observed, such as shifts in peak positions and variations in population weights. These results highlight the ability of the present approach to reproduce the torsional distributions derived from atomistic simulations.

#### Computational Efficiency

The CG dihedral fitting procedure can also be performed using linear least-squares (LLS), for example, by selecting a maximum frequency of *n* = 6 for the potential and a higher cutoff (e.g., *n* = 12) for fitting the AA and CG energy curves. However, this approach is less efficient, as LLS scales as $O\left({\mathrm{Mn}}^{2}+n^{3}\right)$ ,^59^ whereas the FFT scales as O(Mlog⁡M) and does not depend on the number of retained frequencies. This difference becomes significant when fitting multiple dihedrals, as encountered in biomolecular systems.

## Discussion

The FFT approach proved successful in generating torsional potentials regardless of the multiplicity, symmetry, or structural complexity of the underlying dihedral. It applied to both all-atom and coarse-grained models, despite the fact that these two regimes require fundamentally different strategies to obtain the reference potential-energy profiles. **For AA parametrization**, we conclude that the FFT method can serve as a unified framework that provides a unified alternative to traditional Linear Least Squares fitting for both symmetric Ryckaert–Bellemans–type profiles and asymmetric, highly anharmonic torsions. When the same number of frequency Fourier components is used, the proposed approach yields results comparable to conventional methods such as RB or LLS-general fitting. Its main advantage emerges in more complex cases, where higher-frequency contributions are relevant, and the appropriate truncation is not known a priori, or lower-frequency terms can be neglected due to their insignificant contribution, as the spectral decomposition provides a systematic and more efficient framework for identifying and selecting significant components. Importantly, FFT also provides consistent phase assignments for all Fourier components, enabling the correct treatment of multipath dihedrals irrespective of atom hybridization or local geometry—an aspect that cannot be handled automatically by standard Fourier fitting. Overall, the proposed methodology for AA dihedral fitting combines iterative refinement with amplitude-based spectral filtering to provide a robust and systematic framework for torsional parametrization. Together, these features enable an adaptive parametrization strategy that balances accuracy, efficiency, and transferability. **For CG parametrization**, FFT again yielded torsional potentials capable of reproducing AA-derived energy landscapes through iterative refinement. Because PES scans cannot be easily derived for CG models, Iterative Boltzmann Inversion provided a practical route to generate CG torsional potentials directly from AA and CG distributions.

An advantage of the FFT + IBI workflow is that it can also be applied to AA parametrization in situations where direct MM scans fail—for example, when structural rearrangements during PES scans lead to discontinuities or artificial barriers. In such cases, an AA trajectory can be used as a stable reference, pairing FFT reconstruction with an IBI-style refinement to recover smooth torsional potentials. Across both AA and CG applications, the primary limitation of FFT is not computational but methodological: arbitrarily high frequencies can always reproduce fine structural features of a torsional PES, yet including too many terms may be inefficient or undesirable. In this sense, FFT provides a convergent hierarchy of models whose complexity can be chosen by the user, offering a clearer and more systematic alternative to the ad hoc truncation of traditional Fourier fitting schemes.^2,23^

In the case of the Aβ42 peptide, FFT successfully reproduced the complex multimodal shapes of the AA distributions, with the only limitation being the incomplete resolution of very sharp peaks around 60°, which would require higher Fourier frequencies than the *n*_max_ = 6 limit imposed here. Given the reduced resolution of CG models and the associated computational considerations, it may not always be desirable to include such high-frequency components. Users must balance representational accuracy against performance and model simplicity.

Finally, it is worth noting that recent machine-learning–based parametrization frameworks, such as ParametrizANI, also rely on truncated Fourier representations to fit torsional profiles. While these approaches can efficiently model symmetric torsions, they exhibit limitations when confronted with strongly asymmetric PESs.^60^ In these cases, neither the ML reference PES (ANI-2x) nor the fitted Fourier expansion fully captures the skewed asymmetric wells. By contrast, the FFT representation is free from fixed multiplicity assumptions, and it naturally handles such asymmetric and multifrequency features, providing a more general and systematically convergent method for these challenging torsions.

In summary, we conclude that the FFT framework offers a robust, automated, and highly transferable approach for torsional parametrization across AA and CG models. Its flexibility, phase consistency, and compatibility with iterative refinement make it an attractive alternative to traditional Fourier-based schemes and ML-assisted parametrization pipelines. The method’s generality and ease of integration into existing MD engines underscore its potential to become a broadly applicable tool for next-generation force-field development.

## Supplementary Material



## Data Availability

The data supporting the findings of this study are publicly available at the following GitHub repository: https://github.com/humbertoTFT/FFT_dihedral. The repository contains the main program for AA dihedral FFT-fitting dihefit_fft.py, as well as the structures and topologies of the molecules studied in this work with initial nonfitted topologies and final FFT-fitted dihedral potentials.

## Notes

Additional figures, details of the molecular dynamics parameters and computational settings used in this study are provided in the Supporting Information, as well as detailed instructions for the use of the program dihefit_fft.py (PDF)
