# Supplementary material for: Fast Fourier Transform Enables Automated Parametrization of Complex Dihedral Potentials in All-Atom and Coarse-Grained Force Fields
Source: J Chem Inf Model. 2026 May 19;66(11):6453–67. doi: 10.1021/acs.jcim.6c00123 (PMC13250899; doi:10.1021/acs.jcim.6c00123)
Supplement: Supplementary file 1 [file ci6c00123_si_001.pdf]

Supporting Information

Fast Fourier Transform Enables Automated Parametrization of Complex Dihedral Potentials in All-Atom and Coarse-Grained Force Fields

Humberto T. Flores–Trujillo[1], Guillermo L. Rodríguez–Segura[1], Carlos Amador-Bedolla[2], and Laura Dominguez[1]*

1 Departamento de Fisicoquímica, Facultad de Química, Universidad Nacional Autónoma de México, Ciudad de México 04510, México

2 Departamento de Física y Química Teórica, Facultad de Química, Universidad Nacional Autónoma de México, Ciudad de México 04510, México

Contents

Supplementary Notes

SN1. Molecular Dynamics parameters for MM scans S3

SN2. Determination of pathway weights S4

SN3. Comparison of computational efficiency of FFT and LLS S6

SN4. Usage of script dihefit_fft.py S7

Supplementary Figures

SF1. Dihedral distribution comparison for MS-Z S12

SF2. Dihedral distributions comparison for histatin 5 S13

Supplementary Note 1. Molecular Dynamics Parameters for MM scans

For the MM scans, the Steepest Descent minimization algorithm was used, allowing for 500 steps of relaxation to a minimum energy of 200 kJ/mol/nm.

For the MS-Z simulations, the same simulation method was used in both all-atom (AA) and coarse-grained (CG) models. A 3.5 x 3.5 x 3.5 nm^3 box was constructed, solvated in water, and minimized using Steepest Descent. The system temperature was always maintained at 300 K using the V-rescale thermostat. For the AA system, a 250 ps NVT dynamics was performed, followed by a 1 ns NPT dynamics using a Berendsen barostat. Subsequently, a 1-microsecond production was performed using a Parrinello-Rahman barostat. For the CG simulations, each iteration involved a single equilibrium using a Berendsen barostat for 500 ps, followed by a 200 ns production using a Parrinello-Rahman barostat.

For the Aβ42 peptide simulations, the reference temperature was maintained at 310 K in both cases using the V-rescale thermostat. Again, in both cases, minimization was performed using Steepest Descent. The atomistic structure of the Aβ42 peptide was obtained from the Protein Data Bank server. This structure was solvated in a TIP3P water box, and Na^+ and Cl^- ions were added at a concentration of 0.1 M. After minimization, an NVT equilibrium was established for 100 ps, followed by an NPT equilibrium for 200 ps using a Parrinello-Rahman barostat. Subsequent production was carried out for 1 microsecond using the same barostat. For the CG simulations, each iteration followed the same scheme as that used for the MS-Z.

Supplementary Note 2. Determination of pathway weights

Least Squares formulation

The coefficients w_p defining the contribution of each pathway to the total torsional potential were determined through a constrained least-squares procedure. Given a set of M sampled dihedral angles $\{\phi_k\}$, the target torsional profile $V_{\text{tors}}(\phi_k)$ is approximated as a linear combination of pathway-specific contributions $V_{\text{tors},p}^{\text{FFT}}(\phi_k)$.

$$V_{\text{tors}}(\phi_k) \approx \sum_{p=1}^{P} w_p \, V_{\text{tors},p}^{\text{FFT}}(\phi_k) \tag{1}$$

this problem can be expressed in matrix formulation as

$$\mathbf{y} = \mathbf{A}\mathbf{w} \tag{2}$$

where the matrix and vectors are

$$\mathbf{y} = \begin{bmatrix} V_{\text{tors}}(\phi_0) \\ V_{\text{tors}}(\phi_1) \\ \vdots \\ V_{\text{tors}}(\phi_{M-1}) \end{bmatrix} \quad \mathbf{w} = \begin{bmatrix} w_1 \\ w_2 \\ \vdots \\ w_P \end{bmatrix} \tag{3}$$

$$\mathbf{A} = \begin{bmatrix} V_{\text{tors},1}^{\text{FFT}}(\phi_0) & V_{\text{tors},2}^{\text{FFT}}(\phi_0) & \cdots & V_{\text{tors},P}^{\text{FFT}}(\phi_0) \\ V_{\text{tors},1}^{\text{FFT}}(\phi_1) & V_{\text{tors},2}^{\text{FFT}}(\phi_1) & \cdots & V_{\text{tors},P}^{\text{FFT}}(\phi_1) \\ \vdots & \vdots & \ddots & \vdots \\ V_{\text{tors},1}^{\text{FFT}}(\phi_{M-1}) & V_{\text{tors},2}^{\text{FFT}}(\phi_{M-1}) & \cdots & V_{\text{tors},P}^{\text{FFT}}(\phi_{M-1}) \end{bmatrix}. \tag{4}$$

Here, the $\mathbf{y}$ contains the target torsional energies, $\mathbf{A}$ is the design matrix whose columns correspond to pathway-specific contributions, and $\mathbf{w}$ contains the unknown coefficients.

The optimal weights are obtained by minimizing the squared residual

$$\min_{\mathbf{w}} \|\mathbf{A}\mathbf{w} - \mathbf{y}\|^2 \tag{5}$$

subject to the normalization constraint

$$\sum_{p=1}^{P} w_p = 1 \tag{6}$$

which ensures that the combined potential preserves the overall energy scale.

Low energy weighting

To account for the fact that low-energy regions of the torsional profile are sampled more frequently in molecular dynamics simulations, a weighted least-squares formulation can be employed. In this case, the objective function becomes

$$\min_{\mathbf{w}} \sum_{k=0}^{M-1} \mu_k \left(V_{\text{tors}}(\phi_k) - \sum_{p=1}^{P} w_p V_{\text{tors},p}^{\text{FFT}}(\phi_k) \right)^2 \tag{7}$$

Where the weighting factors μ_k are defined as

$$\mu_k = \exp\left[-0.2 \sqrt{V_{\text{QM}}(\phi_k)} \right] \tag{8}$$

The constrained (weighted) least-squares problem was solved using standard numerical linear algebra routines. In practice, the normalization constraint was enforced either explicitly or by reducing the dimensionality of the problem through elimination of one variable.

Supplementary Note 3. Comparison of computational efficiency of FFT and LLS.

The CG dihedral fitting procedure can alternatively be performed using linear least-squares (LLS). In this case, the torsional potential can be represented using a limited number of Fourier terms (e.g., $n = 6$), while a higher frequency cutoff (e.g., $n = 12$) may be used to fit the AA and CG energy profiles. This approach removes the need for explicit frequency selection but increases the computational cost of the fitting procedure.

The computational complexity of LLS scales as $O(Mn^2 + n^3)$,[1] where M is the number of sampled dihedral points and n is the number of Fourier terms. In contrast, the fast Fourier transform (FFT) scales as $O(M \log M)$ and does not depend explicitly on the number of retained frequencies.

To estimate the computational cost, we consider a representative CG dihedral fitting scenario. For each dihedral angle, three independent fits are required: one for the final torsional potential and two for the AA and CG energy profiles. Assuming $M = 360$ points per profile, this results in M_{tot} = 1080 data points per dihedral. For biomolecular systems, the number of dihedral angles scales with system size. A protein with B residues contains approximately $B - 3$ backbone dihedral angles, leading to a large number of independent fitting procedures.

Table SN3-1 presents an approximate comparison of the number of operations required by FFT and LLS for different values of M.

M	FFT	LLS
36	186	41,472
360	3,060	274,752
1,080	10,800	793,152
10,000	132,877	7,215,552
100,000	1,660,964	72,015,552
1,000,000	19,931,569	720,015,552

Table SN3-1. Approximate number of operations computed for FFT and LLS methods.

Supplementary Note 4. Implementation Details and Usage Instructions

Code and examples are publicly available at the following GitHub repository: https://github.com/humbertoTFT/FFT_dihedral.

Main script for AA dihedral fit:

- dihefit_fft.py

In order to run the dihefit_fft.py script correctly, the following software requirements must be met:

Software requirements

Linux OS

Gromacs:

- Gromacs 2021.x

Python:

- Python 3.x
- Numpy
- Matplotlib

Gaussian

- Gaussian16

Input Files

- Molecule coordinates (.gro)
- Molecule topology (.itp)
- Parameters file (.info)

Usage

The program dihefit_fft.py runs in three stages:

1. Coordinates and topology for the molecule, as well as a parameter file, are taken to produce a Gaussian16 input file for QM optimization. The output file is the provided as input for the second stage.
2. Optimization output file, as well as parameters file, are taken to produce a Gaussian16 input file for QM dihedral scan.
3. Dihedral scan output file is taken to perform MM dihedral scan with Gromacs 2021.x. Then FFT iterative fit is performed. Final molecule topology file with dihedral potential for best fit is written.

Input parameters file

The input parameters file (params.info) contains all the necessary information to generate QM optimization and dihedral scans files, as well as run the FFT-fitted algorithm. It also describes the molecule dihedral topology based on the atoms index number. In figure SN4-1 we show the structure of molecule CFBN.

The principal axis ***j-k*** of the dihedral angle ***i-j-k-l*** is formed by two carbon atoms with index numbers 1 and 2. In the directive "axis" of parameters.info file the indexes should be written as shown in figure SN4-2. Atoms bonded to these atoms should be written in the directives "j_bonders" (for atoms bonded to ***j*** atom of dihedral ***i-j-k-l***) and "k_bonders" (for atoms bonded to ***k*** atom of dihedral ***i-j-k-l***).

The number of pathways to be used in the optimization procedure is at the user´s discretion. If we wish to include all pathways of dihedral the indexes 3,4,5 (not necessarily in order) should be written in the directive "j_bonders", as well as indexes 6,7,8 in the directive "k-bonders". Only one pathway can be considered including, for example, the number 4 in "j_bonders" and 7 in "k_bonders".

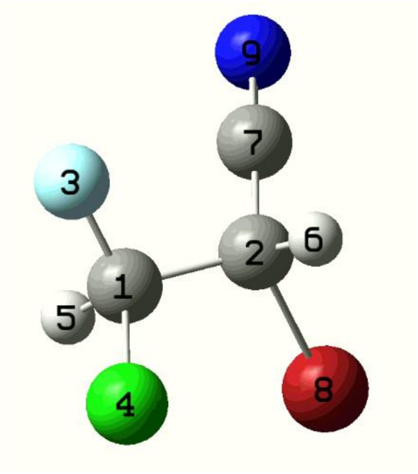

Figure SN4-1. Molecule structure of example molecule CFBN with index numbers.

The "rotation" parameter defines the angular step size (in degrees) employed in the torsional scan. This value determines the resolution of the sampled dihedral energy profile. For instance, a value of 10 corresponds to a uniform sampling every 10° along the 0–360° range (The input file will exclude the last point according to FFT periodicity requirements).

```
# Dihedral parameters
axis = 1 2
j_bonders = 4 3 5
k_bonders = 7 8 6
rotation = 10

# QM parameters
charge = 0
multiplicity = 1
method = wb97xd
basis = 6-31g(d,p)
memory = 2000
nproc = 16

# MM parameters
ff = gromos
lj_fudge = 0.5
qq_fudge = 0.5
min_run = yes
min_nst = 500
min_dt = 0.001
min_emtol = 200
```

Figure SN4-2. Basic input parameters for parameters.info file.

QM parameters: The parameters listed in this section define the quantum mechanical setup used for geometry optimization and torsional energy scans, including the total charge, spin multiplicity, level of theory, basis set, and computational resources. These parameters are user-defined and may be adjusted depending on the system under study and the desired level of accuracy. The values provided here correspond to those used in the present work and are given as a representative example.

MM parameters: The parameters in this section define the molecular mechanics parameters employed for the torsional scans. This includes the choice of force field (ff), scaling factors for Lennard–Jones (lj_fudge) and electrostatic (qq_fudge) interactions, as well as the minimization settings (min_run, min_nst, min_dt, and min_emtol) used to relax the system at each sampled dihedral configuration. These parameters can be adjusted by the user depending on the system and desired level of accuracy; the values reported here correspond to those used in the present work.

Stage 1:

Minimum parameters required:

```
-crd (Molecule coordinates file in Gromacs format)
-top (Molecule .itp topology file in Gromacs format)
-name (User defined name for the working folder and files. It
must not contain spaces)
-file (File with parameters for the program)
```

NOTE: The dihedral angle to be optimized must be turned off in the input topology file.

Run the following command in order to generate the Gaussian16 input file for optimization.

```
python dihefit_fft.py -crd molecule.gro -top molecule.itp -name
MOL -file parameters.info
```

A folder will be generated with the name:

- MOL_dihe

Inside that folder File generated:

- MOL_opt.inp

This optimization input file should be run un Gaussian16. It could be modified to meet QM user requirements. After finishing it should generate either of the two following output files:

- MOL_opt.out
- MOL_opt.log

Stage 2:

Output file after QM optimization should be put in folder MOL_dihe as MOL_opt.log or MOL_opt.out. Then run again the command:

```
python dihefit_fft.py -crd molecule.gro -top molecule.itp -name
MOL -file parameters.info
```

This will generate the file:

- MOL_scan.inp

This dihedral scan input file should be run un Gaussian16. It could be modified to meet QM user requirements. After finishing it should generate either of the two following output files:

- MOL_scan.out
- MOL_scan.log

Stage 3:

Output file after QM dihedral scan should be put in folder MOL_dihe as MOL_scan.log or MOL_scan.out. Then run again the command:

```
python dihefit_fft.py -crd molecule.gro -top molecule.itp -name MOL -file parameters.info
```

The previous command will execute the program with default parameters.

```
-maxf = 6 (Maximum frequency allowed)
-th = 0.98 (R2 score threshold)
-fini = 1 (Minimum number of F frequencies to be tested)
-fend = 6 (Maximum number of F frequencies to be tested)
-iter = 20 (Number of refinement iterations for frequencies set)
```

These options can be modified by the user to meet their requirements. For example, to allow a maximum frequency of 4, a minimum R^2 value of 0.99, allowing just 10 iterations, the command should be:

```
python dihefit_fft.py -crd molecule.gro -top molecule.itp -name MOL -file parameters.info -maxf 4 -th 0.99 -iter 10
```

The final molecule topology file produced with FFT-fitted dihedral potentials will be:

- MOL_dihefit-FFT.itp

Use flag `-h` or `--help` to display help.

```
python dihefit_fft.py --help
```

Supplementary Figures

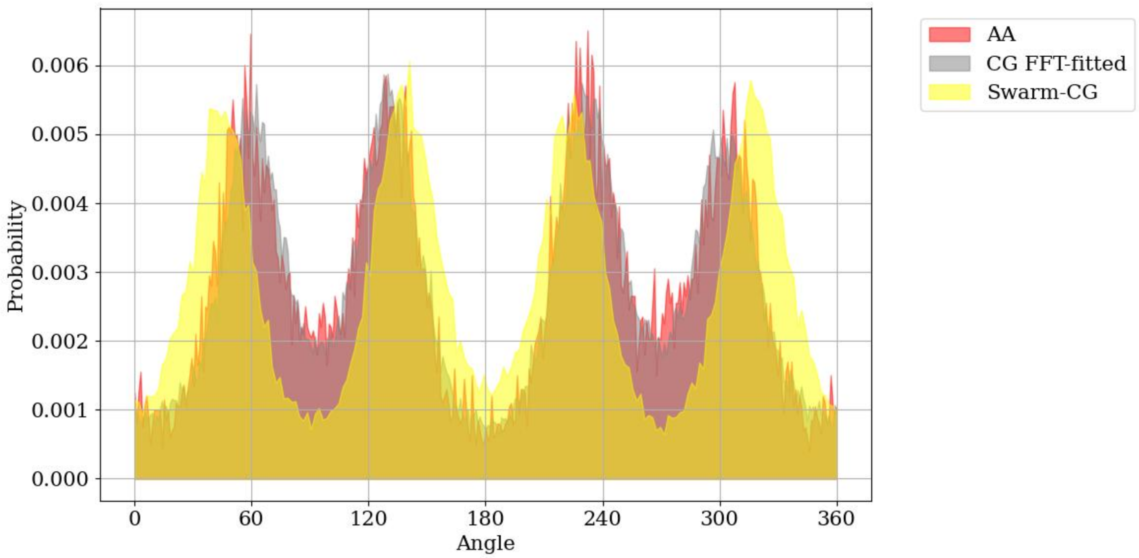

Figure SF1. Distribution for dihedral angle in MS-Z in all-atom (red), and the coarse grain model with FFT-derived potentials (gray) and Swarm-CG (yellow).

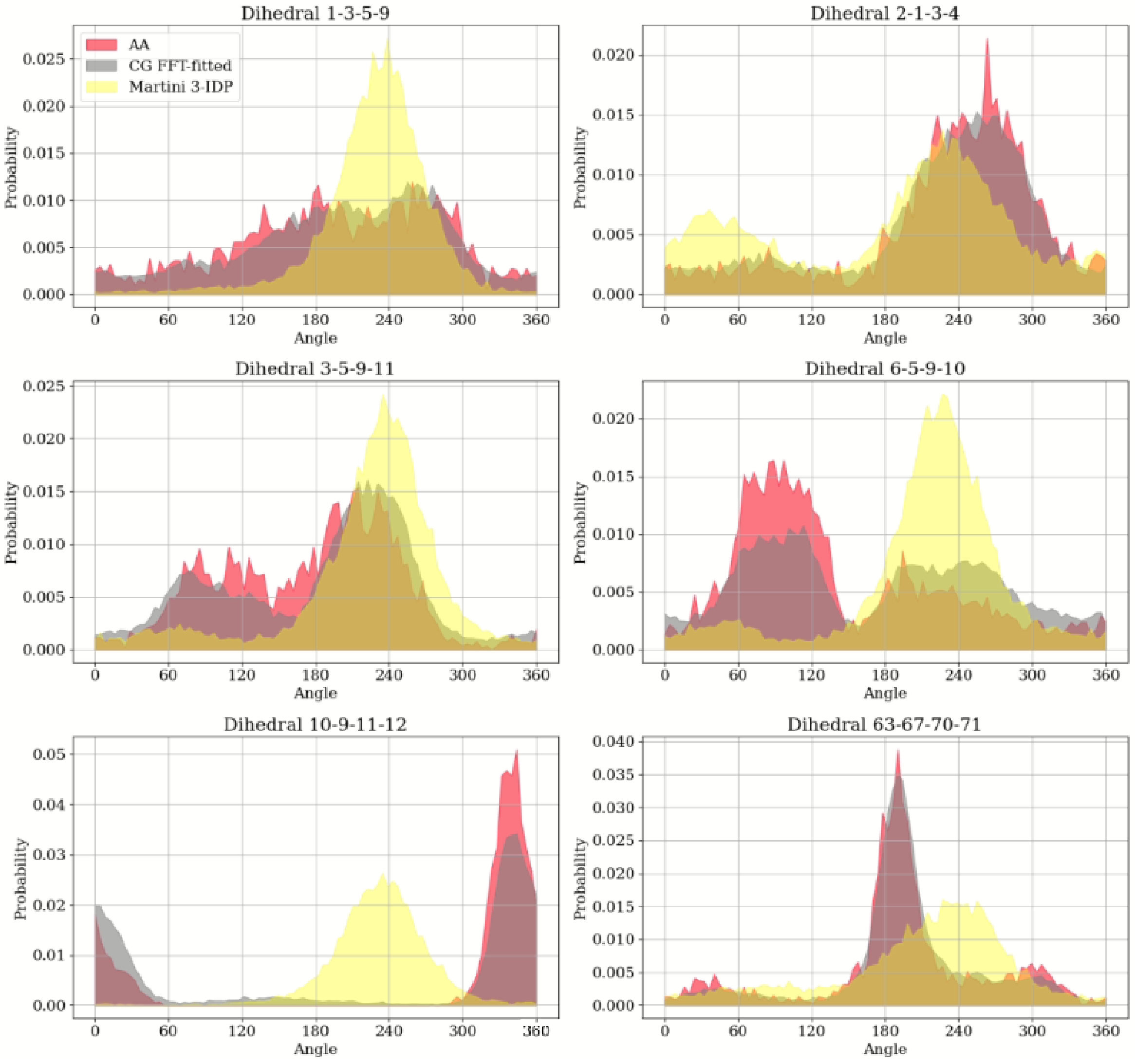

Figure SF2. Distributions for some dihedral angles in Histatine 5 in all-atom (red), and the coarse grain model with FFT-derived potentials (gray) and Martini 3-IDP (yellow).

References

1. Golub, G. H.; Loan, C. F. V., Matrix Computations, 4th; Johns Hopkins University Press: Baltimore, 2013.
